# Phylogenetic and paleobotanical evidence for late Miocene diversification of the Tertiary subtropical lineage of ivies (*Hedera* L., Araliaceae)

**DOI:** 10.1186/s12862-017-0984-1

**Published:** 2017-06-22

**Authors:** V. Valcárcel, B. Guzmán, N. G. Medina, P. Vargas, J. Wen

**Affiliations:** 1 Department of Biology (Botany), Universidad Autónoma de Madrid, Madrid, Spain; 2 Department of Biodiversity and Conservation, Real Jardín Botánico, CSIC, Madrid, Spain; 3 Department of Botany, Faculty of Science, University of South Bohemia, Ceske Budejovice, Czech Republic; 4 Department of Botany/MRC 166, Smithsonian Institution, Washington, DC USA

**Keywords:** Eastern and western Mediterranean, Tertiary refuge, Centrifugal dispersal, Climate-driven spatial speciation

## Abstract

**Background:**

*Hedera* (ivies) is one of the few temperate genera of the primarily tropical Asian Palmate group of the Araliaceae, which extends its range out of Asia to Europe and the Mediterranean basin. Phylogenetic and phylogeographic results suggested Asia as the center of origin and the western Mediterranean region as one of the secondary centers of diversification. The bird-dispersed fleshy fruits of ivies suggest frequent dispersal over long distances (e.g. Macaronesian archipelagos), although reducing the impact of geographic barriers to gene flow in mainland species. Genetic isolation associated with geographic barriers and independent polyploidization events have been postulated as the main driving forces of diversification. In this study we aim to evaluate past and present diversification patterns in *Hedera* within a geographic and temporal framework to clarify the biogeographic history of the genus.

**Results:**

Phylogenetic (biogeographic, time divergence and diversification) and phylogeographic (coalescence) analyses using four DNA regions (*nr*ITS, *trn*H-*psb*A, *trn*T-*trn*L, *rpl*32) revealed a complex spatial pattern of lineage divergence. Scarce geographic limitation to gene flow and limited diversification are observed during the early-mid Miocene, followed by a diversification rate increase related to geographic divergence from the Tortonian/Messinian. Genetic and palaeobotanical evidence points the origin of the *Hedera* clade in Asia, followed by a gradual E-W Asian extinction and the progressive E-W Mediterranean colonization. The temporal framework for the E Asia - W Mediterranean westward colonization herein reported is congruent with the fossil record. Subsequent range expansion in Europe and back colonization to Asia is also inferred. Uneven diversification among geographic areas occurred from the Tortonian/Messinian onwards with limited diversification in the newly colonized European and Asian regions. Eastern and western Mediterranean regions acted as refugia for Miocene and post-Miocene lineages, with a similar role as consecutive centers of centrifugal dispersal (including islands) and speciation.

**Conclusions:**

The Miocene Asian extinction and European survival of *Hedera* question the general pattern of Tertiary regional extinction of temperate angiosperms in Europe while they survived in Asia. The Tortonian/Messinian diversification increase of ivies in the Mediterranean challenges the idea that this aridity period was responsible for the extinction of the Mediterranean subtropical Tertiary flora. Differential responses of *Hedera* to geographic barriers throughout its evolutionary history, linked to spatial isolation related to historical geologic and climatic constraints may have shaped diversification of ivies in concert with recurrent polyploidy.

**Electronic supplementary material:**

The online version of this article (doi:10.1186/s12862-017-0984-1) contains supplementary material, which is available to authorized users.

## Background

**Fig. 1:**
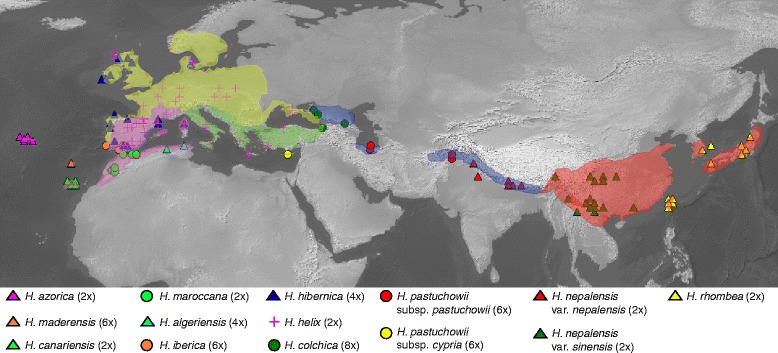
Geographic distribution of the samples of *Hedera* included in the phylogeographic study. In parenthesis level of ploidy. Coloured envelopes delimit the five biogeographic regions recognised in *Hedera*

*Hedera* (ivies) is an Old World root-climber genus that extends from N Africa to Europe and S Asia [1–4]. The main diagnostic characters for species identification and recognition are morphological features from foliar trichomes and vegetative leaves [5]. However, ploidy level and geographic distribution provide fundamental information for species delimitation [5, 6] (Fig. 1). For example, ploidy level was essential for the identification of two morphologically similar species that were traditionally considered as the same species (*H. helix*: 2×, *H. hibernica*: 4×) [7], or for the segregation of two N African endemics (*H. algeriensis*: 4×, *H. maroccana*: 2×) [8–10]. In addition, geographic isolation helped distinguish two closely related species (*H. iberica*, SW Iberian Peninsula; *H. maderensis,* Madeira) [11], or disclose incipient speciation processes (*H. nepalensis* var. *nepalensis*, Himalaya; var. *sinensis*, E & SE China) [12, 13]. The combination of morphological and cytogenetic variation together with geographic information, help characterize 12 species (14 taxa): 6 diploid species (3 island endemics), 2 tetraploid species, 4 hexaploid taxa (2 island endemics), and 1 octoploid species (Fig. 1). The numerous island endemics (five) and the strong geographic structure detected in the DNA sequence variation [4, 11, 14–16] are interpreted as an imprint of the geographic barriers in the diversification process of *Hedera*. However, the endozoochorus dispersal syndrome of ivies, mainly mediated by birds [17, 18], together with the winter ripping of their fleshy fruits when food is scarce for animals, suggests that small geographic obstacles might not be such effective barriers to gene flow.

Ivies usually occupy shaded and humid understories of temperate and subtropical woodlands and riparian vegetation. However, they can also live in extremely dry environments such as fully sun-exposed rocks [19, 20]. Not all species are equally tolerant to both deep shaded and drought environments. Indeed, whereas *H. helix* occurs under the above-described contrasted environments, other species show very strict environmental requirements. For example, *H. iberica* is restricted to warm humid places (“Canuto”) in southern Iberia [21], where remnants of the Tertiary flora also occur [22, 23]. Another example is *H. canariensis* that only occurs in humid, warm and shaded understories of the Macaronesian subtropical woodland ‘Laurisilva’ [24]. The fact that some species display the subtropical affinity that characterized the Asian Palmate group of the Araliaceae while others show a strict temperate tendency, may be suggesting that climate might have also contributed to the speciation in *Hedera*.

Different phylogenetic studies have described a very complex evolutionary history for this genus [4, 6, 13–16, 25]. The origin of the *Hedera* clade has been estimated in the Oligocene in Asia during the deep radiation of the Asian Palmate group of the Araliaceae [25, 26]. In previous phylogeographic studies conducted within *Hedera*, Asia was also suggested as the ancestral area of the extant species of ivies [16, 25]. It has been hypothesized that extinctions in Asia together with Mediterranean colonization and diversification have led to the present distribution. In this scenario, current Asian species of the genus would be the result of a re-colonization from the Mediterranean [15].

In this study we aim to reconstruct ancient and recent diversification patterns in *Hedera* under the working hypothesis that geographic barriers have determined the main patterns of diversification by promoting speciation. Under this working hypothesis, divergence events in *Hedera* would be expected to occur preferentially between areas after a colonization event. The resulting geographic isolation would have led to a strong geographic structure in the genetic variation. To evaluate this hypothesis we analyzed three plastid DNA spacer/intron regions (*trn*H-*psb*A, *trn*T-*trn*L, and *rpL*12) and the nuclear ribosomal ITS (nrDNA) region. First, a *nr*ITS dated phylogeny was reconstructed and used as a starting point to conduct biogeographic and diversification analyses. Phylogenetic results based on *nr*ITS data were examined together with those obtained from the phylogeographic analysis of the plastid dataset. To achieve our ultimate goal of clarifying the biogeographic and phylogeographic history of *Hedera*, the following specific objectives were addressed, the: (1) study of the Mediterranean and Asian areas using a geographically-balanced targeted sampling, (2) reconstruction of past and present diversification patterns, and (3) evaluation of the importance of geographic barriers in promoting isolation.

## Methods

### Taxon sampling and sequencing

#### Phylogenetic sampling

A phylogenetic-based study was performed to provide a temporal context to conduct the biogeographic and diversification analyses for reconstructing the evolution of the *Hedera* clade*.* Wide sampling of outgroup is needed for biogeographic inferences when the nodes of interest approximate the root of the ingroup tree because node’s estimates partly rely on the optimization of their stems [27]. Therefore, all the generic-lineages of the Asian Palmate group have been included, as well as the putative sister-group of the Asian Palmate group (the *Aralia*-*Panax* group). The phylogenetic-based analyses (biogeographic, diversification and divergence age analyses) used the *nr*ITS region because: (1) there is a large number of available sequences of Araliaceae, (2) it is more variable than the fastest evolving plastid regions and provides more resolved tree topologies, (3) it better complements the evolutionary history of the genus where nuclear and plastid incongruence has been previously reported due to hybridization [15, 25], and (4) main diagnostic characters in the taxonomy of *Hedera* come from foliar trichomes and trichomes are genetically controlled by nuclear genes [28, 29]. In any case, all the analyses were also done with the plastid dataset used for the phylogeographic study (see below) but not included in the study because the lack of branch support may have resulted in inaccurate interpretations of the biogeographic, divergence age and diversification results. The *nr*ITS dataset included 34 samples representing 12 species of *Hedera,* 44 of the other 20 generic-lineages of the Asian Palmate group, and 12 other Araliaceae genera (Additional file 1). *Harmsiopanax ingens* was used as the outgroup. All the 90 *nr*ITS sequences were obtained from previous studies [6, 15, 25, 26] and downloaded from GenBank (http://www.ncbi.nlm.nih.gov, Additional file 1).

#### Phylogeographic sampling

A phylogeographic study was conducted to reconstruct the geographic pattern of genetic diversity within *Hedera*, including 153 samples representing the 12 species (14 taxa) recognised (Fig. 1 and Additional file 2). The number of samples per species varied between 5 and 40, except for *H. algeriensis* (endemic to N Algeria and N Tunisia) for which only two samples were available. Sampling effort was more intensive on the two most widespread species, leading to the inclusion of 40 samples of the European *H. helix* and 32 of the Asian *H. nepalensis*. Samples were selected to represent the whole geographic range of each species with an emphasis on the areas considered as Tertiary refugia both in the Mediterranean [30] and in China [31]. To investigate the geographic origin of *Hedera*, *Kalopanax septemlobus* was included as the outgroup (Additional file 2) [25]. Additionally, to evaluate the potential impact of the uncertainty on the sister group of *Hedera* [25], six other Asian Palmate genera were also included (Additional file 2).

Three plastid DNA regions were analyzed (*rpL*32, *trnH-psbA* and *trnT-trnL*) for this part of the study. The primers used for the amplifications were as follows: (1) trn a and trn b for *trnT-L* spacer [32], (2) rpL32F and *trn*L(UAG) for the *rpL32* intron [33], and (3) *trn*HR and *psb*AF for the *trnH-psbA* spacer [34]. Amplifications and sequencing protocols followed Valcárcel et al. [15] for the *trnT-trnL* region, Mitchell et al. [26] for the *trnH-psbA* spacer and Shaw et al. [33] for the *rpL*32 intron. As a result 270 sequences were newly generated in this study (76 for *rpL*32, 89 for *trn*T-*trn*L, and 111 for *trn*H-*psb*A). The three plastid DNA regions of *K. septemlobus* were taken from Li et al. [35] and downloaded from GenBank (http://www.ncbi.nlm.nih.gov), as well as for the other six genera of Araliaceae included. The sampling for the *trn*T-*trn*L spacer was completed by the addition of 44 sequences from our previous phylogeographic studies [15, 16]. Three DNA matrices were compiled using only *Kalopanax* as the outroup: *trn*T*-trn*L (134 samples, 89 new sequences), *trn*H*-psb*A (112 samples, 111 new sequences), and *rpL*32 (77 samples, 76 new sequences). Alignments were carried out with MUSCLE [36] followed by manual revision in Geneious v9.0.5 (http://www.geneious.com). Sequences were concatenated into a fourth matrix with the program Sequence Matrix [37], only including samples with the three DNA regions sequenced (66 samples). A fifth matrix was additionally built to check for the impact of the different rootings of the *Hedera* network on the geographic interpretations, including 65 samples of *Hedera* plus 7 different genera of Araliaceae.

### Phylogenetic-based analyses

#### Divergence age estimates

Divergence age estimates were inferred from the *nr*ITS matrix through a relaxed molecular clock implemented in Beast v.1.7.5 [38]. The substitution rate variation was modeled using an uncorrelated lognormal distribution and a Birth-Death process was applied to model speciation. The best evolutionary model for each of the DNA regions was selected by jModeltest setting a threshold of 3 ∆AIC (Additional file 3) [39]. The analyses were run in the absence of topological constraints, except for the calibration nodes. Two MCMC analyses were run for 100 million generations sampled every 10,000 generations. Convergence, mixing and effective sample size (ESS) of model parameters were assessed using Tracer 1.5 [40]. Samples from the two independent runs were pooled after removing a 25% burn-in using Log Combiner 1.7.5 [38]. Trees were summarized in a maximum clade credibility (MCC) tree obtained in TreeAnotator 1.7.5. Seven leaf macrofossils and two pollen grain fossils have been recorded in *Hedera* (Table 1). The taxonomy of ivies is mainly based on foliar trichomes that are not well preserved in fossils. Also, pollen grains do not show any morphological variation between the extant species of ivies [3]. Therefore, certainty on the phylogenetic placements of these fossils is limited. Only the oldest fossil found (Oligocene, Table 1) can be placed with certainty as a calibration point at the stem of *Hedera*, as inferred from the age recovered for the lineage of *Hedera* in previous studies [between 36.6 Mya and 51.55 Mya; 25]*.* However, we decided not to use this node as a calibration point in the final analyses because the stem of *Hedera* represents an uncertain node in the phylogeny [25]. Instead, two previous divergence time estimates obtained from plastid DNA were employed as secondary calibration points [25]. This secondary calibration approach is more conservative since the age estimates in which it is based were obtained from a fossil-based calibration with a certain placement of fossils on robust nodes [25]. Accordingly, the crown groups of the Asian Palmate and *Hedera* clades were set as normal distributions of 72.55 ± 9.0 Myr and 7.65 ± 3.5 Myr, respectively. Calibration accuracy was tested by comparing divergence times herein estimated to the ages of reliable *Hedera* fossils [41–50] (Table 1). A second Beast analysis was performed using the oldest fossil of *Hedera* as the minimum age for the stem of *Hedera* to double check the posterior age recovered for the crown of *Hedera* in the secondary calibration estimate.

**Table 1:** Fossil records of *Hedera* (entries arranged in chronological order)

Taxon	Locality	Biogeographic region	Age	Size class	Reference
*Hedera* sp.	Pongsan, Korea	E Asia	Oligocene (39.9–23 Mya)	Macrofossil	[44, 46]
*Hedera* cf. *multinervis*	Abkhazia, Georgia	W Asia	Miocene	Macrofossil (leaf)	[47]
*Hedera* cf. *multinervis*	Vegora, Greece	E Mediterranean	Miocene	Macrofossil (leaf)	[41, 48]
*Hedera orbiculata*	Silesia, Poland	Europe	Langhian (16.0–11.6 Mya)	Microfossil (pollen)	[41]
*Hedera* cf. *multinervis*	Cerdanya, Spain	W Mediterranean	Tortonian (11.6–11.3 Mya)	Macrofossil (leaf)	[42, 45]
*Hedera* sp.	Iberian Peninsula	W Mediterranean	Upper Miocene (11.7–5.3 Mya)	Microfossil (pollen)	[45]
*Hedera* cf. *helix*	Italy	W Mediterranean	Messinian (7.2–5.3 Mya)	Macrofossil (leaf)	[49]
*Hedera* sp.	NW Portugal	Europe	Pliocene (5.3–2.6 Mya)	Microfossil (pollen)	[50]
*Hedera orbiculata*	Thuringia, Germany	Europe	Piacenzian (3.6–2.6 Mya)	Macrofossil	[43]

#### Biogeographic range estimation

**Fig. 2:**
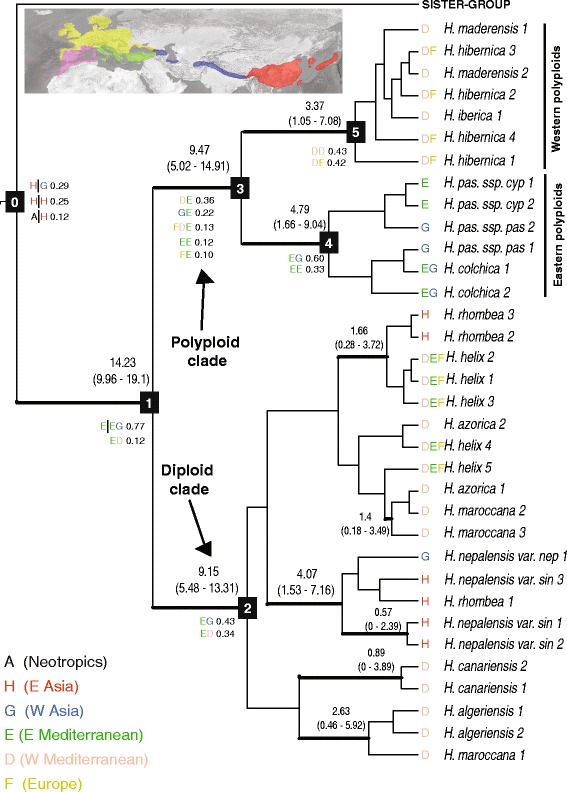
Beast Maximum Clade Credibility chronogram of the *Hedera* clade from the *nr*ITS dataset. Mean ages and 95% CI are only represented for clades with >0.95 Posterior Probability support. Nodes of interest are labelled. Ancestral areas and estimated probabilities obtained from the Lagrange multi-tree analysis are provided only for the nodes of interest. The complete Asian Palmate group MCC tree including the 90 sequences of Araliaceae is shown in Additional file 5. Branch lengths were modified from the original tree (Additional file 5) to better fit the biogeographic results

Estimation of spatial patterns of geographic diversification in *Hedera* was conducted using a model-based likelihood method (Lagrange) and the *nr*ITS dataset. A Dispersal-Extinction-Cladogenesis analysis was performed over multiple trees using a script provided by Richard Ree (*pers. com.*). A multi-tree approach was essential to account for the impact of phylogenetic uncertainty on the ancestral range estimate of the stem group of *Hedera*, due to its ambiguous sister-group relationship [25]. For this purpose, 1000 post-burnin trees from the *nr*ITS Beast analysis were randomly selected with the R-package ape [51] and used as input trees for Lagrange. The geographic range of the Araliaceae was divided into eight regions based on floristic endemicity with special emphasis on *Hedera* distribution: (A) tropical Africa, (B) Neotropics, (C) Australia, (D) western Mediterranean region (including Macaronesia, hereafter W Mediterranean), (E) eastern Mediterranean region (hereafter E Mediterranean), (F) Europe, (G) western Asia (hereafter W Asia), and (H) eastern Asia (hereafter E Asia). The codification of areas for each sample is provided in Additional file 1. In range constraints, adjacency of areas was allowed only between areas that share the edge (i.e., between W Mediterranean and Europe, and between E Mediterranean and W Asia). Maximum range size was set to two areas. Ranges allowed in the analysis included all possible combinations within those imposed by adjacency and maximum range size. Because we were only interested in recovering the early evolution of *Hedera*, results were only computed for the most internal nodes of *Hedera* with support (posterior probability (PP) >0.95; Fig. 2). Particularly, eight nodes were analyzed: the stem and crown groups of the *Hedera* clade (nodes 0 and 1; Fig. 2), two main clades (nodes 2 and 3; Fig. 2) and two main subclades (nodes 4 and 5; Fig. 2). The output file obtained from the Lagrange analysis of 1000 trees was read and parsed with a new R script herein designed for parsing multi-tree Lagrange results (Additional file 4). This script makes automatic parsing Lagrange multi-tree results, which simplifies the process of summarizing results saving time. The results were summarized as the mean of probabilities estimated by Lagrange for the posterior trees analyzed. The specific ancestral areas of the descendants of a given node are provided when a congruent biogeographic pattern is consistently recovered over the multi-trees analyzed (e.g.*,* nodes 0 and 1; Fig. 2). If the estimated ancestral areas of the descendants of a given node *i* resulted in incongruent biogeographic patterns when the multi-trees results were analyzed together, only the ancestral areas of node *i* are provided with no specification to the descendant lineages. The ancestral area for a given node *i* is assumed to be the combination of the ancestral areas estimated for its descendants. For example, if the most probable biogeographic patterns for a given node are (1) E|D at a mean probability of 0.39, (2) D|E at a mean probability of 0.16, and (3) ED|D at a mean probability of 0.14; then, a simplification of the results are shown by providing the ancestral area for the node as ED with a mean probability of 0.69.

#### Diversification analyses

The 6500 post-burnin Beast trees were pruned to only consider the clade of *Hedera* and one tip per species, except for non-monophyletic species for which one tip per species-lineages was kept (18 tips, 14 taxa). We decided to only analyze the clade of *Hedera* because the independent analysis of a particular clade is recommended to isolate its diversification pattern from the heterogeneous diversification patterns of other clades in the phylogeny [52]. The stem of *Hedera* was included in the analysis because ignoring long branches before crown nodes may result in inaccurate interpretations of the diversification pattern within the crown group [53, 54]. The resulting 6500 post-burnin pruned trees were used as the inputs for the diversification analyses. The log-transformed number of extant taxa was plotted against time (LTT plot) for the 6500 post-burnin pruned trees using the R-package ape [51]. Fitness to speciation models with one, two or three diversification rates was also tested as implemented in the R-package LASER [55]. The best evolutionary model was selected for each of the 6500 post-burnin pruned trees based on the AIC using a ∆AIC of 4. Two contingency table tests with one dimension and two levels were performed over the results of the 6500 trees using chi-square goodness of fit [56] in R [57]: (1) number of trees with constant vs. variable rate models and (2) number of trees with Yule two rates vs. Yule three rates variable models.

The Phylogenetic Diversity (PD) index of Faith [58] measures the length of evolutionary pathways that connect a given set of taxa as the sum of branch lengths connecting taxa in a given area. In this study, Faith’s index was used to account for the amount of PD of *Hedera* represented in each of the five major endemicity areas delimited for ivies in the biogeographic analyses (W Mediterranean, Europe, E Mediterranean, W Asia, E Asia). The aim of this analysis was to evaluate whether the most species-rich endemicity areas also hold the greatest evolutionary diversity. Faith’s PD was estimated as implemented in the R-package Picante [59]. To assess if regions have significantly higher or lower PDs than at random expectations for a given number of species, PDs were calculated over the 1000 randomized posterior trees used for the Lagrange analysis but pruned to only consider the clade of *Hedera* and one tip per species-lineages. Two-tailed test was used to compare the observed PDs to the null distribution of the 1000 random replicates (significance level of 0.05).

Network reconstructions based on the coalescence [58] analysis of plastid haplotypes were performed on the five-plastid DNA matrices. Statistical Parsimony (SP) was applied as implemented in TCS 1.13 [60]. The 95% probability limit of parsimonious connections was applied and gaps were coded as missing data. Predictions from coalescent theory were applied to deals with homoplasy [61, 62]. To test the hypothesis that interior and tip haplotypes are equally frequent we used a contingency table test with one dimension and two levels using chi-square goodness of fit [56] in R [57].

## Results

### Estimates of divergence times

The early divergence age estimates of the *Hedera* clade (Fig. 2, Additional file 5) fit the timing set by the fossil record (Table 1). The two main clades of *Hedera* (diploid and polyploid) diverged in parallel during the late Miocene (9.47 / 9.15 Mya, 5.02–14.91 / 5.48–13.31 Mya 95% CI; nodes 2 / 3, Fig. 2), five million years after the crown age divergence of the *Hedera* clade in the early-mid Miocene (14.23, 9.96–19.1 Mya 95% CI; node 1; Fig. 2). The divergence time estimate for the eastern polyploid subclade is 4.79 Mya (1.66–9.04 Mya 95% CI; node 4), whereas 3.37 Mya (1.05–7.08 Mya 95% CI; node 5) is recovered for the western polyploid subclade. The diploid clade displays a large basal polytomy that prevents from any further divergence time or biogeographic estimates (Fig. 2). The posterior estimates on the calibration node 1 lay outside the bound of the calibration, which is interpreted as low influence of this calibration prior on the posterior. The MCC Beast tree obtained after removing this secondary calibration point and including the oldest fossil of *Hedera* as the calibration point at the stem of *Hedera* revealed similar posterior node estimates (Additional file 6).

### Ancestral range inference

Results from the multi-tree Lagrange analysis (1000 posterior random trees) plotted on the *nr*ITS MCC Beast tree of *Hedera* posit Asia as the most likely ancestral area for the stem of the genus (|H or |G, *P* = 0.66; node 0; Fig. 2). The most probable range inferred for the crown group of *Hedera* is the combination of the E Mediterranean and W Asia (*P* = 0.77; node 1). This result suggests that an extinction event occurred in E Asia between *Hedera’s* stem and crown along with a dispersal event to the E Mediterranean. Subsequently, two equally plausible biogeographic scenarios are inferred for the diploid clade (from node 1 to 2; Fig. 2): (1) persistence in the ancestral area (EG, *P* = 0.43; node 2) or (2) W Asian extinction and dispersal to the W Mediterranean (ED, *P* = 0.34; node 2). The most probable biogeographic scenario for the polyploid clade (from node 1 to 3; Fig. 2) is the E Mediterranean persistence (E, *P* = 0.93; node 3), W Asian extinction (≠ G, *P* = 0.71), and the W Mediterranean colonization (D, *P* = 0.49). The W Mediterranean is recovered as part of the ancestral area for the divergence of the western polyploid subclade (node 5; D, *P* = 0.85; Fig. 2), whereas divergence of the eastern polyploid subclade may have occurred in the E Mediterranean and W Asia (EG, *P* = 0.66; node 4) or in the E Mediterranean (EE, *P* = 0.33).

### Diversification analyses

**Fig. 3:**
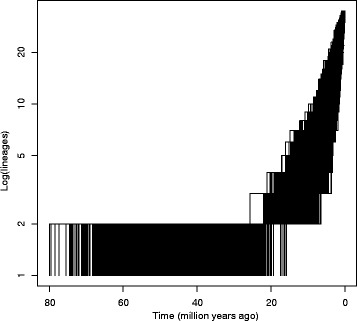
Lineage through time plots of the 6500 posterior pruned trees of the *Hedera* clade from the *nr*ITS dataset

Rate variable models (Yule two rates and Yule three rates) are more frequently selected as the best fitting speciation process than constant ones (pureBirth and birth-death) among the 6500 posterior pruned trees (4003 vs. 2497, respectively; *U* = 359.5319, *p*-value <0.001; Additional file 7). The Yule two rates model estimates an initial diversification of 0.04 per lineage per unit of time (−0.14–0.23 95% CI) that increased to 0.29 (0.024–0.55 95% CI) at around 7.82 Mya (5.94–9.71 95% CI). The equally probable Yule three rates (*U* = 0.89, *p*-value = 0.4) recovered an initial diversification of 0.04 (−0.29–0.37 95% CI) that increased to 0.94 (−2.96–4.45 95% CI) at around 10.19 Mya (7.42–12.97 95% CI) and decreased to 0.36 (−1.11–1.84 95% CI) at 3.20 Mya (0.35–6.05 95% CI). The LTT plot of the 6500 posterior pruned trees describes a flat initial diversification rate period followed by an ever-increasing diversification pattern from the Miocene onwards for the *Hedera* clade (Fig. 3). This graphical representation is congruent with the recovery of Yule two rates and Yule three rates as the best fitting speciation models. The E Mediterranean and W Asia are the only regions that reveal greater PDs than expected at random for the given number of species (Table 2). The largest value of Faith’s PD index is estimated for W Mediterranean (72.05, Table 2), while the smallest value is computed for E Asia (32.54). The remaining three main biogeographic areas display similar low values of PD index (Table 2).

**Table 2:** Summary of different diversity indicators in *Hedera* according to the five biogeographic regions recognized

Biogeographic region	N_spp_	Ploidy levels	Interior Hps	Tip Hps	Observed PD	Lower PD	Upper PD
W Mediterranean	8	2×, 4×, 6×, 8×	6	7	72.05***	73.51	75.12
E Mediterranean	3	2×, 6×, 8×	0	2	44.95*	38.73	41.77
Europe	2	2×, 4×	2	3	43.53^*n.s.*^	31.29	34.64
W Asia	3	2×, 6×, 8×	1	2	44.34***	38.92	41.98
E Asia	2	2×	1	4	32.54***	38.31	41.47

**Fig. 4:**
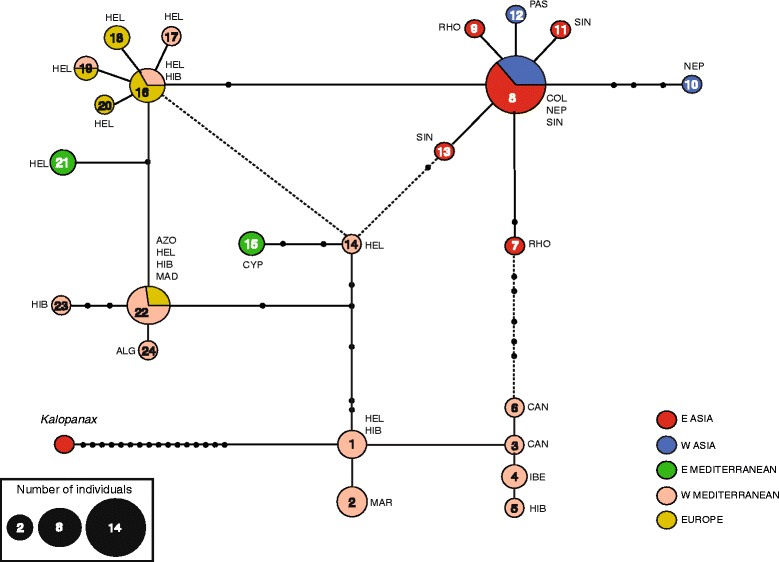
Phylogeographic network of the *Hedera* plastid matrix (*rpL*32, *trn*H-*psb*A, *trn*T-*trn*L) including *Kalopanax septemlobus* as outgroup. Haplotype numbering is according to Additional file 8. Circle dimensions are proportional to the number of samples displaying each haplotype as indicated at the bottom. Lines indicate a single nucleotide substitution and dots (●) represent extinct or not-detected haplotypes. Dashed lines indicate ambiguities resolved under predictions of the Coalescent Theory [65]. Abbreviation names of the taxa displaying each haplotype are as follows: ALG, *H. algeriensis*; AZO, *H. azorica*; CAN, *H. canariensis*; COL, *H. colchica*; CYP, *H. pastuchowii* subsp. *cypria*; HEL, *H. helix*; HIB, *H. hibernica*; IBE, *H. iberica*; MAD, *H. maderensis*; MAR, *H. maroccana;* NEP, *H. nepalensis* var. *nepalensis*; PAS, *H. pastuchowii* subsp. *pastuchowii*; RHO, *H. rhombea*; SIN, *H. nepalensis* var. *sinensis*

*Nspp:* Number of species, *HPs: * Haplotypes, Observed *PD*: Faith's Phylogenetic Diversity Index [48, 52], Lower PD and Upper PD: Lower and upper bounds of the null distribution of the empirical randomization of PD. Level of significance is indicated as followed: ***P≤0.001, *P≤0.05, n.s. *P* >0.05. Interior and tip haplotypes are according to Fig. 4

### Phylogeographic networks

Twenty-four haplotypes are detected within *Hedera* when the three-plastid DNA regions are concatenated (Fig. 4, Additional file 8). The highest number of haplotypes is detected in the Mediterranean region with 15 haplotypes, 57% of them exclusive to E Asia. Seven haplotypes are detected in Asia, 67% of them exclusive. Finally, five haplotypes occur in Europe, with only the 2 of them exclusive. The most frequent haplotype (Hp 8) is detected in 14 Asian samples (9 from E Asia and 5 from W Asia) of two species (*H. colchica*, *H. nepalensis*), followed by two Euro-Mediterranean haplotypes (Hp 22: 8 Mediterranean samples, 3 European; Hp 16: 4 European samples, 2 Mediterranean). Comparable geographic patterns of haplotype diversity are obtained when individual plastid matrices are analyzed (Additional files 9, 10 and 11). The W Mediterranean is consistently recovered as the geographic region with the highest number of haplotypes (*rpL*32: 6 Hps, *trn*H-*psb*A: 5 Hps, *trn*T-*trn*L: 7 Hps; Additional files 9, 10 and 11). The area with the second highest number of haplotypes is E Asia according to *trn*H*-psb*A (4 Hps; Additional file 10) and *trn*T*-trn*L (8 Hps*;* Additional file 11) while Europe according to *rpL*32 (4 Hps; Additional file 9).

The number of interior haplotypes is 10 (6 unambiguous, 29 samples; 4 ambiguous, 4 samples) while 14 are tip haplotypes (23 samples). Five of the six unambiguous interior haplotypes are from the W Mediterranean (5) and Europe (2) while only one from Asia (Table 2, Fig. 4). The mean number of sequences per haplotype varies from 4.8 for unambiguous interior haplotypes and 1.64 for tip haplotypes. Also the ratio of single-sample haplotyes (singleton) vs. non-singleton haplotypes varies between 0.2 for interior haplotypes and 0.6 for tip haplotypes. Interior haplotypes are more frequent than tip ones (*U* = 14.087, *p*-value <0.001). This result together with the application of Templeton’s rules allows us to resolve the network uncertainties (Fig. 4).

Limited taxonomic congruence is detected in the haplotype network, with the widespread Euro-Mediterranean *H. helix* and *H. hibernica* and the E Asian *H. rhombea* displaying between two and eight unrelated haplotypes (Fig. 4 and Additional file 8). However, strong geographic structure and environmental affinity is detected (Fig. 4). The W Mediterranean haplotypes are scattered through the network, whereas the majority of the European and Asian haplotypes are organized in two star-like groups. The Euro-Mediterranean and Asian start-like groups are connected to each other through their most widespread haplotypes (Hp 16 and Hp 8, respectively; Fig. 4). The haplotypes of all the samples included from the southwest Mediterranean are connected with no missing haplotype needed (hereafter “relict SW Mediterranean haplotypes”). The independent analysis of each plastid region consistently recovers the group of relict SW Mediterranean haplotypes and the Asian star-like group connected to the widespread Euro-Mediterranean star-like group (Additional files 9, 10 and 11).

The *Hedera* network is connected to *Kalopanax* (outgroup) through the relict SW Mediterranean Hp 1 with 16 missing haplotypes needed for connection. Similarly, a relict SW Mediterranean haplotype is connected to an Asian outgroup when the seven genera of Araliaceae are included (Additional file 12). Connection to outgroup slightly differs when plastid datasets are analyzed independently (Additional files 9, 10 and 11). The biogeographic connection to the outgroup is inferred through a widespread Euro-Mediterranean haplotype in *rpL*32 (Additional file 9) or through E Asian haplotypes in *trn*H-*psb*A (Additional file 10) and *trn*T-*trn*L (Additional file 11). The Taiwanese sample that connects to the outgroup in the *trn*H-*psb*A network (Additional file 10) is recovered as a tip haplotype when the three DNA regions are analyzed together (Hp 7; Fig. 4).

## Discussion

### Uneven geographic diversification of *Hedera*: E Asia as both ancestral and sink area

Eastern Asia is inferred as the most likely ancestral area for the *Hedera* clade (Fig. 2), which is congruent with the E Asian location of the oldest fossil record of *Hedera* (Table 1). This geographic context is also consistent with the fact that all the remaining 20 generic-lineages of the Asian Palmate group occur in E Asia. Indeed, tropical and subtropical SE Asian environments are inferred to be the center of diversification of the Asian Palmate group and some of its genera [26, 63–69]. This E Asian placement of the ancestor of the temperate *Hedera* clade is congruent with the Asian origin of numerous lineages of the flora of Europe [70–72]. Westward dispersal and E Asia extinction appear to have occurred early in the evolution of the clade of *Hedera* leading to an initial divergence during the Lower-Middle Miocene in W Asia and the E Mediterranean (Fig. 2). The fossil record also supports the timing of this westward migration, since reliable fossils of *Hedera* are found for the first time in W Asia (Georgia) [47] and the E Mediterranean (Greece) [48] during the Lower-Middle Miocene (Table 1). The two early descendant lineages (polyploid and diploid clades) persisted in the E Mediterranean with a probable and independent W Mediterranean colonization coupled with W Asian extinction in the polyploid clade. The low diversification rate found for this period in the *Hedera* clade (r_0_ = 0.03–0.04, >10.19 Mya - >7.82 Mya; Fig. 3, Additional file 7) is better explained by a high extinction rate in Asia. This Asian extinction scenario supports the previous biogeographic hypothesis of Asian extinction and Mediterranean differentiation of *Hedera* [15, 16], but challenges the general pattern of Tertiary regional extinction of temperate genera in Europe and survival in Asia or North America [73–75]. The E-W gradual extinction in Asia is not only supported by the biogeographic analysis (Fig. 2), but also by the high number of missing haplotypes that are needed to connect the relict SW Mediterranean group to the Asian outgroup in the network (Fig. 4, Additional file 12). Soon after the westward migration and Asian extinction, a diversification rate increase is detected for *Hedera* starting in the Tortonian/Messinian (10.19 or 7.82 Mya; Fig. 3, Additional file 7), when ivies were already established at both sides of the Mediterranean as inferred from biogeographic reconstruction (Fig. 2) and fossil record (Table 1). The increase in aridity that synchronously started across the whole basin by that time [76–78] has been proposed to be responsible for the extinction of most of the sub-tropical Tertiary plants that previously inhabited this area [79–81]. The diversification rate increase of *Hedera* is intriguing since it places the intensification of diversification under an arid climate, which is an unpredicted scenario for a group of plants that need humid environments. Confinement to wet habitats in locally isolated environments could have promoted isolation and thus speciation for those elements of the sub-tropical Tertiary flora that were also tolerant to arid conditions. This could be the case of ivies that are considered part of the dry sub-tropical Tertiary flora [69, 82, 83]. Indeed, certain Mediterranean traits have been observed in *Hedera* species: low stomatal density primarily on leaf underside (Virginia Valcárcel *pers. obs.*), high density of indumentum on young shoots (Hugh A. McAllister *pers. obs.*), and flowering only in sun-exposed branches (Pablo Vargas *pers. obs.*). Independent colonization events from the Mediterranean to other areas of Europe and independent back colonizations to Asia are inferred coinciding with the highest diversification periods (10.19–3.20 Mya or 7.82 Mya - present, Additional file 7). Interestingly, the intense diversification that accompanied this post-Miocene geographic range expansion did not occur evenly in all areas. As a result, an unbalanced diversity pattern is currently observed with the ancestral area (E Asia) operating as a sink region for the diversity of extant ivies (Table 2) and the distant Mediterranean basin acting as its source of re-colonization.

### E and W Mediterranean as refugia in the Miocene and Pliocene

Two main regions are considered as Tertiary and Quaternary refugia for temperate and subtropical plant lineages in Eurasia, the Mediterranean basin [30] and SW China [73, 84]. The fact that Pliocene and Pleistocene extinctions were more intense in Europe than in Asia [75] has been explained by several factors, such as climatic and topographic changes, complex geological history, or the antiquity of the flora in Asia ([84], but see [85]). As a whole, there is a greater diversity of temperate genera in Asia than in Europe [73]. After a thorough full sampling of *Hedera* in the Mediterranean and Asian Tertiary refugia (Fig. 1), the Mediterranean region is not only still inferred as the main center of diversification for ivies [16] but also as a cumulative lineage refugium. The E and W Mediterranean are interpreted as consecutive refugia with differential roles in the evolution of the genus. Whereas the E Mediterranean acted as the refugium for *Hedera* during the dramatic changes of the Miocene, the W Mediterranean is identified as a more recent refugium for ivies during the climatic changes that shaped the Mediterranean flora from the Pliocene onwards. Our biogeographic analysis suggests that the E Mediterranean was part of the ancestral area back during the early differentiation of *Hedera* in the Miocene (nodes 2–3; Fig. 2), which is also supported by the time of the first fossils of *Hedera* in the Mediterranean that are from the eastern side [48] (Table 1). This pattern reveals that the E Mediterranean was a phylogenetic center of diversification in the past that served as a Miocene refugium for the ancestors of the lineages of extant ivies. This scenario is also supported by the fact that the E Mediterranean has a greater Faith’s PD than expected by chance (Table 2). The reason why the E Mediterranean currently shows low diversity might be due to higher extinction rates in this area and higher levels of differentiation in the W Mediterranean. Currently, the E Mediterranean only has three species, three ploidy levels, two-plastid tip haplotypes and a relatively low PD index (45, Table 2). The limited diversity of extant ivies in the E Mediterranean contrasts the results of some studies showing this side of the basin as a center of diversification for different groups of organisms [86–92]. From the ancestral refugium in the E Mediterranean a centrifugal dispersal resulted in the colonization of the W Mediterranean in the first place and the back colonization of W Asia in more recent times. A similar centrifugal dispersal from the E Mediterranean has been described for *Anthemis* also in the Miocene [93]. Despite the relevance of the E Mediterranean in the early evolution of the genus, the W Mediterranean is the region that accounts for the highest diversity in ivies. Indeed, the W Mediterranean displays the greatest number of extant species (eight), ploidy levels (four), plastid haplotypes (13, Additional files 7), and Faith’s PD (72, Table 2). This is consistent with the pattern observed in the phylogeographic study where the W Mediterranean samples are scattered through the network displaying internal and tip haplotypes both in relict and derived groups (Fig. 4). Also, seven out of the 10 internal haplotypes and six out of the 13 tip haplotypes are widespread or unique to western Mediterranean samples (Fig. 4). All these results support the W Mediterranean as a secondary center of diversification [6], which might also be the reason for this area to have the greatest PD index but with this index lower than the one expected by chance (Table 2). All sources of evidence agree with the W Mediterranean as a source area of dispersal in the post-Miocene colonization of Europe and E Asia. Indeed, the star-like organization of the Asian and Euro-Mediterranean haplotypes (Fig. 4) suggest a recent and fast colonization from the W Mediterranean.

The relative importance of the E and W Mediterranean regions as refugia for the Mediterranean flora varies among groups [see 30 for details] with a general trend towards considering the W Mediterranean as the main Tertiary refugium (33 cases vs. 19 in E Mediterranean) [30]. It seems reasonable to assume that the role of the E Mediterranean as an ancestral refugium might have been overlooked because the more recent diversification processes might have erased footprints of earlier events, as in the case of *Hedera*.

### Geographic isolation related to new habitats as the driver for *Heder*a evolution

The pattern of endemicity in *Hedera*, with 10 endemic taxa (five island endemics) and three widespread species (*H. helix, H. hibernica* and *H. nepalensis*), can only be explained by a key role of geographic isolation in speciation. However, geographic isolation seems less likely for a bird-dispersed fleshy-fruited plant group, like *Hedera*, due to the expected long-distance dispersability of endozoochorous dispersal syndromes mediated by birds [94]. Successful dispersal is supported by the occurrence of three species of *Hedera* in three archipelagoes of Macaronesia. Nevertheless, complex and different geographic patterns of genetic variation have been suggested for fleshy-fruited plants [14, 95, 96]. Evidence points to additional external factors that may alter the efficiency of dispersal mediated frugivory, such as site availability (spatial limitation) [97]. The geographic structure inferred from the biogeographic and phylogeographic reconstructions of *Hedera* is not spatially or temporarily constant. Geographic isolation is interpreted for the recent past of the genus from the geographic congruence of main clades diverged during the Pliocene (nodes 4, 5; Fig. 2). In contrast, back to the early evolution of *Hedera*, diversification occurred within the same geographic area (nodes 1–3, Fig. 2). This may be explained by a combination of historical and contemporary geological and ecological factors. The early divergence of the genus that led to the diploid and polyploid clades most likely took place in the E Mediterranean and W Asia during the Lower-Middle Miocene (Fig. 2, Table 1). By that time the Arabian microplate collided with Eurasia resulting in a major change to a colder climate and the uplift of E Mediterranean basin (~16 Mya) [98]. This may have promoted regional or habitat-dependent isolation that cannot be observed at the scale of biogeographic analyses. The independent range expansion to the W Mediterranean that occurred in the diploid and polyploid clades before the end of the Miocene (Fig. 2) does not seem to result in the observation of any divergence pattern either. During that period the Mediterranean region was under seasonal sub-tropical climate, which may have been crucial for *Hedera* establishment and dispersal across the basin, hindering geographic isolation. Given that the diploid and polyploid clades persisted in the E Mediterranean, the similarity in the diversification pattern detected could be related to the short compressional event that took place in Eastern Aegean 9–8 Mya [99] that may have promoted a secondary contact between the two clades. Such contact provided a reliable context for the inter-lineage hybridization already proposed in *Hedera* [6, 15] that might have eventually resulted in genome duplication [100]. Also, it provides a likely explanation for the incongruence detected between plastid and nuclear results [15] (cf. Figs. 2 and 4). The evolution of the diploid clade remains unclear, whereas a vicariant event is found for the divergence of the polyploid clade leading to the eastern and western polyploid subclades. Interestingly the time of lineage differentiation for eastern polyploids appears to be older than that of the western one, which was unexpected given that the W Mediterranean basin is much older [98]. This may be the result of a geographic filter due to the closer proximity of the E Mediterranean to the ancestral range of ivies (E Asia).

## Conclusions

The consequences of the geological changes occurred in the Mediterranean area during Lower-Middle Miocene were dramatic for the Mediterranean Tertiary plant lineages [79, 81]. However, the subtropical *Hedera* clade survived and diversified in an arid and relatively constant climate. Indeed, the diversification rate increase of ivies since the Tortonian/Messinian under the increasing aridity in the Mediterranean, suggests that a climate-driven spatial limitation (i.e. habitat availability) may have enabled geographic speciation. Cumulative Miocene and post-Miocene refugia are detected in E and W Mediterranean, respectively, that also acted as consecutive dispersal centers.

## Additional files

Additional file 1: List of the studied material included in the phylogenetic-based analyses. Localities, geographic area codification and GenGank accession numbers of the nuclear Internal Transcribed Spacer are provided. Areas abbreviation are as follows: A, Neotropics; B, Tropical Africa; C, Asutralia: D, W Mediterranean; E, E Mediterranean; F, Europe; G, W Asia; H, E Asia. Papers of reference are provided as superscript as follows: (1) Li et al. [35], (2) Mitchell et al. [26], (3) Mitchell et al. [66], (4) Valcárcel et al. [25], (5) Vargas et al. [7], and (6) Valcárcel et al. [16]. (DOCX 141 kb)

Additional file 2: GenBank accession numbers of the studied material included in the phylogeographic study. (DOCX 171 kb)

Additional file 3: Summary of DNA sequences variation and evolutionary models best fitting the nuclear and plastid matrices. (DOCX 41 kb)

Additional file 4: R script for parsing Lagrange results from multi-tree analyses. (R 22 kb)

Additional file 5: Beast Maximum Clade Credibility chronogram of the *nr*ITS dataset of Araliaceae with the secondary calibration approach. Legend: Mean ages and 95% CI are only represented for clades with >0.5 Posterior Probability support. (PDF 255 kb)

Additional file 6: Beast Maximum Clade Credibility chronogram of the *nr*ITS dataset of Araliaceae with the fossil calibration approach. Mean ages and 95% CI are only represented for clades with >0.5 Posterior Probability support. (PDF 236 kb)

Additional file 7: Fitness to speciation models. Summary of the results of the fitness to speciation models estimated from 6500 posterior pruned trees of the *Hedera* clade. N indicates the number of trees that recover a given evolutionary model. Mean values and 95% CI are provided for AIC and each of the parameters of the models. (DOCX 49 kb)

Additional file 8: List of the haplotypes detected in the samples included in the phylogeographic study. Taxa name and general distribution is provided. Locality and voucher is specified for each sample as well as the number of haplotype detected individually for the three regions (*rpL*32, *trnH-psbA*, *trnT-trnL*). Last column indicates the number of haplotype when combining the three-plastid regions *rpL32, trnH-psbA* and *trnT-trnL* (HP^3^). (DOCX 106 kb)

Additional file 9: *Hedera* phylogeographic network obtained from the Statistical Parsimony analysis of the *rpL*32 plastid region. Haplotype numbering is according to Additional file 8. Circle dimensions are proportional to the number of samples displaying each haplotype. *Kalopanax septembolus* is used as outgroup. (PDF 96 kb)

Additional file 10: *Hedera* phylogeographic network obtained from the Statistical Parsimony analysis of the *trn*H-*psb*A plastid region. Haplotype numbering is according to Additional file 8. Circle dimensions are proportional to the number of samples displaying each haplotype. *Kalopanax septembolus* is used as outgroup. (PDF 93 kb)

Additional file 11: *Hedera* phylogeographic network obtained from the Statistical Parsimony analysis of the *trn*T-*trn*L plastid region. Haplotype numbering is according to Additional file 8. Circle dimensions are proportional to the number of samples displaying each haplotype. *Kalopanax septembolus* is used as outgroup. (PDF 108 kb)

Additional file 12: *Hedera* phylogeographic network obtained from the Statistical Parsimony analysis of the three-plastid regions (*trn*H-*psb*A, *trn*T-*trn*L, *rpL*32) and using seven Araliaceae genera as outgroup. Circle dimensions are proportional to the number of samples displaying each haplotype. Abbreviation names of the taxa displaying each haplotype are as follows: ALG, *H. algeriensis*; AZO, *H. azorica*; CAN, *H. canariensis*; COL, *H. colchica*; CYP, *H. pastuchowii* subsp. *cypria*; HEL, *H. helix*; HIB, *H. hibernica*; IBE, *H. iberica*; MAD, *H. maderensis*; MAR, *H. maroccana;* NEP, *H. nepalensis* var. *nepalensis*; PAS, *H. pastuchowii* subsp. *pastuchowii*; RHO, *H. rhombea*; SIN, *H. nepalensis* var. *sinensis.* (PDF 188 kb)

## Abbreviations

My: Million years
Mya: Million years ago
PD: Phylogenetic index
PP: Posterior probability
