# Supplementary material for: Phylogenetic and paleobotanical evidence for late Miocene diversification of the Tertiary subtropical lineage of ivies (Hedera L., Araliaceae)
Source: BMC Evol Biol. 2017 Jun 22;17:146. doi: 10.1186/s12862-017-0984-1 (PMC5480257; doi:10.1186/s12862-017-0984-1)
Supplement: Supplementary file 1 — List of the studied material included in the phylogenetic-based analyses. Localities, geographic area codification and GenGank accession numbers of the nuclear Internal Transcribed Spacer are provided. Areas abbreviation are as follows: A, Neotropics; B, Tropical Africa; C, Asutralia: D, W Mediterranean; E, E Mediterranean; F, Europe; G, W Asia; H, E Asia. Papers of reference are provided as superscript as follows: (1) Li et al. [35], (2) Mitchell et al. [26], (3) Mitchell et al. [66], (4) Valcárcel et al. [25], (5) Vargas et al. [7], and (6) Valcárcel et al. [16]. (DOCX 141 kb) [file 12862_2017_984_MOESM1_ESM.docx]

| **Sample** | **Area** | **Accession** |
| --- | --- | --- |
| *Aralia cordata* Thunb.: Japan, Honshu, *A. Soejima 1072* (US) | AH | GU054649^1^ |
| *Aralia elata* (Miq.) Seem.: China: Chongqi, Jinfoshan, *J. Wen 8221* (US) | AH | GU054685^1^ |
| *Aralia finlaysoniana* (Wall. ex G.Don) Seem.: China: Yunnan, *J. Wen 10571* (US) | AH | GU054665^1^ |
| *Aralia finlaysoniana* (Wall. ex G.Don) Seem.: China: Yunnan, *J. Wen 10571* (US) | AH | GU054665^1^ |
| *Aralia racemosa* L.: U.S.A.: Wisconsin, *J. Wen 7291*–*1* (US) | AH | GU054615^1^ |
| *Aralia soratensis* Marchal.: Bolivia, *M.* *Nee &* *J.* *Wen 53878* (US) | AH | KF591480^4^ |
| *Aralia spinosa* L.: USA: North Carolina, Avery, *J. Wen 6251-15* (US) | AH | GU054699^1^ |
| *Aralia stellata* (King) J. Wen: Thailand, *Maxwell02* (US) | AH | KF591481^1^ |
| *Aralia stipulata* Franch.: China: Xizang, Chayu, *J. Wen 9198* (US) | AH | GU054700^1^ |
| *Aralia vietnamensis* Ha.: China: Yunnan, Luchun, *Y.M. Shui 81844* (US) | AH | GU054666^1^ |
| *Brassaiopsis hispida* Seem.: China: Yunnan, *J. Wen 5031* (US) | H | JX106268^2^ |
| *Brassaiopsis ciliata* Dunn: Vietnam: Lao Cai, *Wen 6038* (US) | H | KF591482^4^ |
| *Brassaiopsis glomerulata* (Bl.) Regal: Vietnam: Lao Cai, *J.* *Wen 5839* (US) | H | KF591483^4^ |
| *Brassaiopsis palmipes* Forrest ex W.W. Smith: China: Yunnan, *J.* *Wen 6512* (US) | H | AY304805^3^ |
| *Chengiopanax fargesii* (*Franch.*) C.B.Shang & J.Y.Huang.: China: Hunan, *J. Wen 9316* (US) | H | GU054651^1^ |
| *Dendropanax arboreus* (L.) Decne. & Planch.: Costa Rica: San Jose, Canton de Acosta, *J. Wen 7045* (US) | AH | GU054692^1^ |
| *Dendropanax caloneurus* (Harms) Merr.: Vietnam: Lao Cai, Sa Pa, *J. Wen 6063-5* (US) | AH | GU054617^1^ |
| *Dendropanax dentiger* (Harms) Merr.: Hunan, China, *J. Wen 9306* (US) | AH | GU054654^1^ |
| *Dendropanax hainanensis* (Merr. & Chun) Chun.: China: Hunan, Mangshan, *Y.F. Deng 16240* (US) | AH | GU054655^1^ |
| *Dendropanax sessiliflorus* (Standl. & A. C. Sm.) A. C. Sm.: Costa Rica: Puntarenas, Canton de Colfito, *J. Wen 7002* (US) | AH | GU054622^1^ |
| *Eleutherococcus senticosus* (Rupr. & Maxim.) Maxim.: China: Beijing, Baihuashan, *J. Wen 8527* (US) | H | GU054610^1^ |
| *Eleutherococus sieboldianus* (Makino) Koidz.: Japan: Chiba-ken, *J. Wen 8538* (US) | H | GU054611^1^ |
| *Fatsia japonica* (Thunb.) Decaisne & Planch.: U.S.A., cult. At Cornell University, *J. Wen 6228* (US) | H | KF591484^4^ |
| *Fatsia polycarpa* Hayata: China: Taiwan, *J. Wen 9391* (US) | H | KF591485^4^ |
| *Gamblea innovans* (Siebold & Zucc.) C.B.Shang, Lowry & Frodin: Japan: Honshu, *A.* *Soejima 1094* (US) | H | JX106274^2^ |
| *Gastonia custispongia* Lam.: Belgium, cult. in National Botanic Garden of Belgium, 19942627 (US) | BC | GU054661^1^ |
| *Harmsiopanax ingens* Philipson: Indonesia: West Papua, *J. Wen 10749*–*3* (US) | H | JX106275^2^ |
| *Hedera algeriensis* Hibberd: (1) Algeria, Kabylie, Azazga | D | AJ131216^5^ |
| *Hedera algeriensis*: (2) Cultivated | D | AJ131217^5^ |
| *Hedera azorica* hort. ex Carrière: (1) Portugal: Azores, Sao Miguel | D | AJ131219^5^ |
| *Hedera azorica*: (2) Portugal: Azores, Pico | D | AJ131218^5^ |
| *Hedera canariensis* Willd.: (1) Spain: Canary Islands, Tenerife, *H. A. McAllister 237HAM* | D | AJ131220^5^ |
| *Hedera canariensis*: (2) Spain: Canary Islands, La Palma | D | AJ131221^5^ |
| *Hedera colchica* (K. Koch) K. Koch: (1) Georgia, Caucasus | DEF | AJ131222^5^ |
| *Hedera colchica*: (2) Georgia, E Caucasus, Telavi | DEF | AJ131223^5^ |
| *Hedera helix* L.: (1) Spain, Málaga, Ronda | DEF | AJ131228^5^ |
| *Hedera helix*: (2) United Kingdom: Scotland, S. Uist, *H. A. McAllister 570HAM* | DEF | AF506078^5^ |
| *Hedera helix*: (3) Spain: Málaga, Ronda, *P. Vargas 5PV97* | DEF | AJ131227^5^ |
| *Hedera helix*: (4) Turkey: Mugla Province, Gocek | DEF | AJ131226^5^ |
| *Hedera helix*: (5) Spain: Huesca | DEF | AF506077^6^ |
| *Hedera hibernica* (hort. ex G.Kirchn.) Bean: (1) Spain, Asturias, La Llamiella, *H. A. McAllister 937HAM* | DF | AJ131229^5^ |
| *Hedera hibernica*: (2) Spain: Huelva, *H. A. McAllister 545HAM* | DF | AF506079^6^ |
| *Hedera hibernica*: (3) Spain, Malaga, Competa, *H. A. McAllister 949HAM* | DF | AJ131231^5^ |
| *Hedera hibernica*: (4) Portugal, Lindoso, Gidache, *H. A. McAllister 925HAM* | DF | AJ131230^5^ |
| *Hedera iberica* (McAllister) Ackerfield & J.Wen: (1) Spain, Cadiz, Los Barrios-Alcala | D | AJ131232^5^ |
| *Hedera maroccana* McAllister: (1) Chefchaouen, path to Bab de Lars, Vargas, P. *152PV00 (MAUAM)* | D | AF506080^4^ |
| *Hedera maroccana*: (2) Marrakech, 31 Km south from Ourika valley, McAllister, H.A. *861HAM (LIV)* | D | AJ131236^5^ |
| *Hedera maroccana*: (3) Tetuan, Rift, Idit 10 Km east from Bou Azzer, McAllister, H.A. *868HAM (LIV)* | D | AJ131235^5^ |
| *Hedera maderensis* K. Koch ex A. Rutherf.: (1) Portugal, Madeira, Parque das Queimadas | D | AJ131234^5^ |
| *Hedera maderensis*: (2) Portugal, Madeira, Funchal, *H. A. McAllister 18HAM* | D | AJ131233^5^ |
| *Hedera nepalensis* K.Koch var. *nepalensis*: (1) India, Kashmir, Manat, *H. A. McAllister 246HAM* | G | AJ131237^5^ |
| *Hedera nepalensis* K.Koch var. *sinensis* Rehder: (1) Vietnam: Lao Cai, *J. Wen 5980* (US) | H | GU05463^1^ |
| *Hedera nepalensis* var. *sinensis*: (2) Vietnam, Fan-si-Pan, Lao Cai province, *H. A. McAllister 895HAM* | H | AJ131238^5^ |
| *Hedera nepalensis* var. *sinensis*: (3) China: Hunan, Xinning, *J. Wen 9278* (US) | H | GU054623^1^ |
| *Hedera pastuchowii* Woronow subsp. *pastuchowii*: (1) Cultivated in USA, Illinois, Cook Co., *J. Wen 7322* (US) | G | GU054609^1^ |
| *Hedera pastuchowii* subsp. *pastuchowii*: (2) Iran, Elburz Mts., *H.A. McAllister* 259HAM | G | AJ131239^5^ |
| *Hedera pastuchowii* Woronow subsp. *cypria* (McAllister) Hand: (1) Cyprus, Limasol, Apsiou | E | AJ131224^5^ |
| *Hedera pastuchowii* subsp. *cypria*: (2) Cyprus, Kakopetria | E | AJ131225^5^ |
| *Hedera rhombea* (Miq.) Bean.: (1) Korea, *K.-O. Yoo s.n.* (US) | H | GU054608^1^ |
| *Hedera rhombea*: (2) South Korea, southern coastline of the Korean Peninsula | H | AJ131241^5^ |
| *Hedera rhombea*: (3) cultivated RNG (418-79-05131BM4316) | H | AJ131240^5^ |
| *Heteropanax fragrans* Seem.: Thailand: Chiang Mai, *J. Wen 7492* (US) | H | JX106276^2^ |
| *Kalopanax septemlobus* (Thunb.) Koidz.: China: Hunan, *J. Wen 9341* (US) | H | GU054645^1^ |
| *Macropanax dispermus* (Blume) Kuntze.: Indonesia: West Java, Bandung, *J. Wen 10137-1* (US) | H | GU054607^1^ |
| *Macropanax maingayi* (C. B. Clarke). Philipson: Malaysia: Langat, *J. Wen 8355* (US) | H | GU054646^1^ |
| *Macropanax rosthornii* (Harms) C. Y. Wu ex G. Hoo.: China: Sichuan, Dujiangyan, *J. Wen 9264* (US) | H | GU054613^1^ |
| *Macropanax undulatus* Seem.: China: Yunnan, *J. Wen 8474* (US); | H | GU054624^1^ |
| *Merrillionapax chinensis* H. L. Li: China: Yunnan, *J. Wen 5065* (US) | H | KF591486^4^ |
| *Merrilliopanax listeri* (King) H.L.Li: China: Yunnan, *J. Wen 5038* (US) | H | JX106277^2^ |
| *Metapanax davidii* (Franch.) J.Wen & Frodin: China: Sichuan, *J. Wen 9266* (US); | H | GU054625^1^ |
| *Metapanax delavayi* (Franch.) J. Wen & Frodin: China: Yunnan, Lufeng, *J. Wen 9146* (US); | H | GU054612^1^ |
| *Oplopanax elatus* (Nakai) Nakai: China: Jilin, Wusong, *J. Wen 5418-11* (US) | AH | GU054662^4^ |
| *Oreopanax globosus* J. Wen, sp. nov.: Peru: Dpto. Pasco, Oxapampa, *J. Wen 8599* (US) | A | GU054693^1^ |
| *Oreopanax liebmannii* Marchal: Mexico, Chiapas, *J. Wen 8744* (US) | A | GU054678^1^ |
| *Oreopanax polycephalus* Harms: Peru: Dpto. Pasco, Oxapampa, *J. Wen 8595* (US) | A | GU054638^1^ |
| *Oreopanax xalapense* (Kunth) Decne. & Planch.: Costa Rica: Heredia, *J. Wen 6934* (US) | A | GU054639^1^ |
| *Panax trifolius* L.: U.S.A.: Maryland, *J. Wen 10099* (US) | AH | GU054701^1^ |
| *Schefflera angulata* (Pav.) Harms: Peru: Dpto. Pasco, *J. Wen 8589* (US) | A | GU054640^1^ |
| *Schefflera arboricola* (Hayata) Merr.: China: Yunnan, Baoshan, *J. Wen 6295* (US) | H | GU054626^1^ |
| *Schefflera heptaphylla* (L.) Frodin: Vietnam: Ninh Binh, Mt. Bavi, *J. Wen 6165* (US) | H | GU054641^1^ |
| *Schefflera heterophylla* (Wall. ex G. Don) Harms: Malaysia: Selangor, *J. Wen 8392* (US) | H | GU054642^1^ |
| *Schefflera hypoleuca* (Kurz) Harms: Thailand: Chiang Mai, Doi Intonon, *J. Wen 7427* (US) | H | GU054643^1^ |
| *Schefflera morototoni* (Aubl.) Maguire: Costa Rica: San Jose, Canton de Pérez Zeledóné, *J. Wen 6952* (US) | A | GU054647^1^ |
| *Schefflera pentandra* (Pav.) Harms: Peru: Dpto. Pasco, *J. Wen 8619* (US) | A | GU054627^1^ |
| *Schefflera subulata* R. Vig.: Malaysia: Selangor, *J. Wen 8396* (US) | H | GU054644^1^ |
| *Sinopanax formosanus* (Hayata) H.L.Li: China:Taiwan, *J. Wen 9390* (US) | H | GU054628^1^ |
| *Tetrapanax papyrifer* (Hook.) K.Koch.: Indonesia: cult. in West Java, *J. Wen 10135* (US) | H | GU054663^1^ |
| *Trevesia lateospina* Jebb: Thailand: Lampang, *J. Wen 7480*-*1* (US) | H | GU054664^1^ |
| *Trevesia palmata* (Roxb. Ex Lindley) Visiani.: (1) China: Yunnan, Yongde, *J.* *Wen 5669* (US) | H | KF591488^4^ |
| *Trevesia palmata* (Roxb. Ex Lindley) Visiani.: (2) China: Yunnan, Hekou, *J. Wen 8460* (US) | H | KF591487^4^ |
| *Trevesia sundaica* Miq.: Indonesia: West Java, *J. Wen10162* (US) | H | KF591489^4^ |
