# Supplementary material for: Phylogenetic and paleobotanical evidence for late Miocene diversification of the Tertiary subtropical lineage of ivies (Hedera L., Araliaceae)
Source: BMC Evol Biol. 2017 Jun 22;17:146. doi: 10.1186/s12862-017-0984-1 (PMC5480257; doi:10.1186/s12862-017-0984-1)
Supplement: Supplementary file 2 — GenBank accession numbers of the studied material included in the phylogeographic study. (DOCX 171 kb) [file 12862_2017_984_MOESM2_ESM.docx]

| **Sample** | ***rpL*32** | ***trnH-psbA*** | ***trnT-trnL*** |
| --- | --- | --- | --- |
| **Outgroup** |  |  |  |
| *Aralia undulata* Hand.-Mazz. | KC456163 | KC456163 | KC456163 |
| *Brassaiopsis hainla* (Buch.-Ham.) Seem. | KC456164 | KC456164 | KC456164 |
| *Eleutherococcus senticosus* (Rupr. & Maxim.) Maxim. | JN637765 | JN637765 | JN637765 |
| *Kalopanax septemlobus* (Thunb.) Koidz. | KC456167 | KC456167 | KC456167 |
| *Metapanax delavayi* (Franch.) J. Wen & Frodin | KC456165 | KC456165 | KC456165 |
| *Panax ginseng* C.A.Mey. | AY582139 | AY582139 | AY582139 |
| *Schefflera delavayi* (Franch.) Harms | KC456166 | KC456166 | KC456166 |
| **Ingroup** |  |  |  |
| *Hedera algeriensis* Hibberd |  |  |  |
| (1) Algeria: Kabylie mountains McAllister, H.A. *838HAM (MA)* | – | KY798759 | AF506093 |
| (2) Tunisia: Ain-Draham, Aldasoro, J.J. *A2890(5) (MA)* | KY798683 | KY798758 | KY798590 |
| *Hedera azorica* (Gand) Carrière |  |  |  |
| (1) Pico, McAllister, H.A. *279HAM (LIV)* | – | – | AF506106 |
| (2) Pico, Cabeco das Cabras, Martínez, J. *216JM04 (MAUAM)* | KY798684 | KY798760 | KY798591 |
| (3) Sao Miguel, Hilliers *s.n. (LIV)* | – | – | AF506107 |
| (4) Sao Jorge, Faja, Martínez, J. *225JM04 (MAUAM)* | KY798686 | KY798762 | KY798593 |
| (5) Faial, Caldeira (Horta), Martínez, J. *212JM04 (MAUAM)* | KY798685 | KY798761 | KY798592 |
| *Hedera canariensis* Willd. |  |  |  |
| (1) Gran Canaria, Valleseco, Vasák, V. *s.n. (BR-SP852826)* | – | KY798765 | – |
| (2) Tenerife, Chinobre, Las Mercedes, Martínez, J. *237HAM (MA)* | – | – | AF506085 |
| (3) La Gomera, Parque Natural de Garajonay, Valcárcel, V. *58VV00 (MA)* | – | – | AF506086 |
| (4) La Gomera, P. N. Garajonay, El Cedro, Vargas, P. *172PV05 (MAUAM)* | KY798687 | KY798763 | KY798594 |
| (5) La Palma, La Galga, el Cubo de la Galga, Valcárcel, V. *66VV04(11) (MAUAM)* | KY798688 | KY798764 | KY798595 |
| *Hedera colchica* (K.Koch) K.Koch |  |  |  |
| (1) Georgia: T'elavi, Zagodeki, McAllister, H.A. *470HAM (LIV)* | – | – | AF506105 |
| (2) Georgia: Lagodekhi, Opred, I. *s.n. M0080097* | KY798691 | KY798768 | KY798598 |
| (3) Georgia: Prov. Krasnodar, between Babuk-Aul and Solokh-Aul, Latschaschvili, J. *s.n. (576348MA)* | – | KY798769 | – |
| (4) Russia: Krasnodar, Krashodarsky Krai, Wen, J. *10358 (US)* | KY798690 | KY798767 | KY798597 |
| (5) Turkey: Rize, near Ikizdere, *Nisa, S. 760SN (MA689076)* | KY798689 | KY798766 | KY798596 |
| *Hedera helix* L. |  |  |  |
| (1) Austria: Baden, Valcárcel, V. *42VV03(2) (MAUAM)* | – | KY798789 | KY798617 |
| (2) Belgium: Brussels, Gossellies, Vargas, P. *114PV03(1) (MAUAM)* | KY798707 | KY798791 | KY798619 |
| (3) Denmark: Jernhatten, Mols, Larsen, K. *275 (MA186341)* | – | – | JQ745317 |
| (4) France: Corsica, Valcárcel, V. *78VV00 (MAUAM)* | KY798703 | KY798784 | KY798612 |
| (5) France: Chizé, Grivet, D. *12HH7 (MAUAM)* | KY798750 | – | KY798627 |
| (6) France: Moint Ventoux, near Malaucena, Valcárcel, V. *155VV01 (MAUAM)* | – | KY798785 | KY798613 |
| (7) France: Pyrenees, Gabas, Portalet pass, Vargas, P. *338PV02(3) (MAUAM)* | KY798704 | KY798786 | KY798614 |
| (8) France: Fontainebleau, Grivet, D. *11HH10 (MAUAM)* | – | KY798799 | – |
| (9) Germany: Wiesbaden, Eberbach Kloster, Vargas, P. *169PV01(1) (MA)* | KY798708 | KY798792 | AF506096 |
| (10) Germany: Bad Reichen hall, Sch. Weissbach, Vargas, P. *386PV02(2) (MAUAM)* | – | KY798793 | KY798620 |
| (11) Greece: Crete, between Vlatos and Ellos, Strovles, Vargas, P. *116PV05 (MA)* | KY798702 | KY798782 | KY798611 |
| (12) Greece: Peloponeso, A. Kalovryta, *Vargas, P. 395PV02(1)bis (MAUAM)* | KY798694 | KY798772 | KY798601 |
| (13) Hungary: Budapest, Pest, Vargas, P. *106PV03(2) (MAUAM)* | – | KY798795 | KY798622 |
| (14) Italy: Ercolano, Vesubio, Vargas, P. *215PV01(2) (MAUAM)* | KY798700 | KY798780 | KY798609 |
| (15) Italy: Godi pass, Scanno, Vargas, P. *217PV01(1) (MAUAM)* | – | – | KY798626 |
| (16) Italy: Sicily, Siracusa, Sortino, Iblei mountain, Herrero, A. *AH1002 (MA)* | KY798701 | KY798781 | KY798610 |
| (17) Moravia: Moravia septenrionalis, distr. Sumperk, in vicinitate pagi Temenice, loco "Pod Lvákem" dicto., Vasák, V. *s.n. (918956BR)* | – | KY798796 | KY798623 |
| (18) Poland: Pogórze Wisnickie foothills, vicinity of Gosprzydowa, Vargas, P. *117PV03(1) (MAUAM)* | KY798709 | KY798794 | KY798621 |
| (19) Spain: Huesca, *Vargas, P. s.n. (MA)* | – | – | AF506098 |
| (20) Spain: Málaga, McAllister, H.A. s.n. *(LIV)* | – | – | AF506090 |
| (21) Spain: Murcia, Aedo, C. *5934CA (MA)* | – | – | JQ745321 |
| (22) Spain: Málaga, Ronda, El Quejigal, Vargas, P. *5PV97 (MA)* | – | – | AF506081 |
| (23) Spain: Granada, near Competa, Salto de Maroma, McAllister, H.A. *953HAM (MA)* | – | – | AF506082 |
| (24) Spain: Almería, Sierra de Gádor, Fondón, Vargas, P. *14PV05(4) (MAUAM)* | – | KY798773 | KY798602 |
| (25) Spain: Granada, Sierra Nevada, Alpujarras, Maza de Lino, Vargas, P. *121PV04 (MAUAM)* | KY798695 | KY798774 | KY798603 |
| (26) Spain: Gerona, Nieto Feliner, G. *4344GN (MA)* | – | – | JQ745316 |
| (27) Spain: Jaén, Cazorla, Valcárcel, V. *16VV02 (MAUAM)* | KY798696 | KY798775 | KY798604 |
| (28) Spain: Madrid, Valle del Paular, Vargas, P. *387PV02(8) (MAUAM)* | KY798697 | KY798776 | KY798605 |
| (29) Spain: Guadalajara, Tamajón, Retiendas, Valcárcel, V. *4VV01 (MAUAM)* | – | KY798777 | KY798606 |
| (30) Spain: Soria, Valcárcel, V. *9VV01 (MAUAM)* | KY798698 | KY798778 | KY798607 |
| (31) Spain: Huesca, Linas de Broto, near Cotefablo pass, Vargas, P. *335PV02(2) (MAUAM)* | KY798749 | – | KY798625 |
| (32) Spain: Menorca, Valcárcel, V. *37VV03(1) (MAUAM)* | KY798699 | KY798779 | KY798608 |
| (33) Spain: Valencia, Castellón, Bellavista, *Guzmán, B. 127BGA04(2)* | KY798710 | KY798798 | KY798624 |
| (34) Switzerland: Murten, Valcárcel, V. *17VV01 (MA)* | KY798706 | KY798790 | KY798618 |
| (35) Turkey: Mugla, Compton, J.A. *s.n. (LIV)* | – | – | AF506091 |
| (36) Turkey: Zonguldak, Ahmetusta pass, Aedo, C. *6519CA (MA)* | – | KY798797 | – |
| (37) United Kingdom: Scotland, S.Uist, S.Glendale, Bagh Mor., McAllister, H.A. *570HAM (MA)* | – | – | AF506097 |
| (38) United Kingdom: Scotland, South west Hebrides, Valcárcel, V. *430VV01 (MAUAM)* | – | KY798788 | KY798616 |
| (39) Ukraine: Crimea, near Yalta, Uchan- Su waterfall, Vargas, P. *116PV03 (MAUAM)* | KY798705 | KY798787 | KY798615 |
| (40) Ukraine: Crimea, Yalta, Valcárcel, V. *427VV01 (MAUAM)* | – | KY798783 | – |
| *Hedera hibernica* (G.Kirchn.) Bean |  |  |  |
| (1) Portugal: Lindoso, McAllister, H.A. *925HAM (MA)* | – | – | AF506109 |
| (2) Portugal: Louso, Serra do Caramulo, Ribeiro, P. *336PR (MAUAM)* | KY798711 | KY798800 | KY798628 |
| (3) France: St Chinian, Vargas, P. *229PV06 (MAUAM)* | KY798716 | KY798805 | KY798633 |
| (4) France: St Andrea Di bozio, Grivet, D. *15HH02 (MAUAM)* | KY798717 | KY798806 | KY798634 |
| (5) United Kingdom: Ireland, Cashel  Vargas, P. *180PV10(1) (MAUAM)* | KY798718 | KY798807 | KY798635 |
| (6) Spain: Asturias, Monte Andorso, Valdés, McAllister, H.A. *937HAM (MA)* | – | – | AF506108 |
| (7) Spain: Granada, near Competa, McAllister, H.A. *949HAM (MA)* | – | – | AF506087 |
| (8) Spain: Huelva, Jabugo, McAllister, H.A. *545HAM (MA)* | – | – | AF506110 |
| (9) Spain: Cádiz, Grazalema, Las Palomas pass, Valcárcel, V. *103VV00 (MAUAM)* | KY798712 | KY798801 | KY798629 |
| (10) Spain: Málaga, between Antequera and Ayora, Valcárcel, V. *8VV02(1) (MAUAM)* | KY798713 | KY798802 | KY798630 |
| (11) Spain: Santander, Bollacín, El Escudo pass, Vargas, P. *127PV01(6) (MAUAM)* | KY798714 | KY798803 | KY798631 |
| (12) Spain: Gerona, Nieto Feliner, G. *4615GN(3) (MA)* | KY798715 | KY798804 | KY798632 |
| (13) United Kingdom: Ireland, Torc Waterfall, Vargas, P. *171PV10(1) (MAUAM)* | KY798719 | KY798808 | KY798636 |
| (14) United Kingdom: Ireland, Glengarriff, Vargas, P. *177PV10(2) (MAUAM)* | KY798720 | KY798809 | KY798637 |
| (15) United Kingdom: Ireland, Crookstown, Vargas, P. *170PV10 (MAUAM)* | KY798721 | KY798810 | KY798638 |
| (16) United Kingdom: Scotland, Drumnadrochit, Vargas, P. *70PV12(1) (MAUAM)* | – | KY798811 | KY798639 |
| (17) United Kingdom: Scotland, Island of Skye, Portree, Vargas, P. *73PV12 (MAUAM)* | – | KY798812 | KY798640 |
| (18) United Kingdom: Scotland, Edinburgh, Vargas, P. *75PV12 (MAUAM)* | – | KY798813 | KY798641 |
| *Hedera iberica* (McAllister) Ackerfield & J.Wen |  |  |  |
| (1) Spain: Cádiz, Los Barrios, Stirling, A.M. *s.n. (MA)* | – | – | AF506088 |
| (2) Spain: Cádiz, Alcalá de los Gazules, "Porto Oscuro", Valcárcel, V. *392VV01(1) (MAUAM)* | KY798722 | KY798814 | KY798642 |
| (3) Spain: Cádiz, Algeciras-Tarifa, "El Bujeo", Sierra de Luna, Valcárcel, V. *399VV01(2) (MAUAM)* | – | KY798815 | KY798644 |
| (4) Spain: Málaga, Sierra Bermeja, Valcárcel, V. *400VV01bis (MAUAM)* | KY798723 | KY798816 | KY798643 |
| (5) Portugal: Monchique Mountains, Segura Zubizarreta, A. *2017 (MA350593)* | – | – | AF506089 |
| *Hedera maderensis* K.Koch ex A.Rutherf. |  |  |  |
| (1) Portugal: Madeira, Funchal, McAllister, H.A. *18HAM (LIV)* | – | – | AF506111 |
| (2) Portugal: Madeira, Das Queimadas Park, Franquinho L.O. *s.n. (LIV)* | – | – | AF506112 |
| (3) Portugal: Madeira, Santa near Achada da Cruz, Valcárcel, V. *05VV08(10) (MAUAM)* | – | KY798817 | KY798645 |
| (4) Portugal: Madeira, Funchal, from Achadas da Cruz to Moniz pass, Vargas, P. 325PV00 (MA654987) | KY798724 | KY798818 | KY798646 |
| (5) Portugal: Madeira, Levada de Casa do Lombo do Mouro, Navarro, C. *CN3395 (MA654727)* | KY798725 | KY798819 | KY798647 |
| *Hedera maroccana* McAllister |  |  |  |
| (1) Morocco: Marrakech, 31 Km south from Ourika valley, Perring, F. *s.n. (MA)* | – | – | AF506092 |
| (2) Morocco: Tetuan, Rift, Idit 10 Km east from Bou Azzer, McAllister, H.A. *868HAM (LIV)* | – | – | AF506083 |
| (3) Morocco: Gurugu Mountain, Vargas, P. *137PV04(7) (MAUAM)* | KY798726 | KY798820 | KY798648 |
| (4) Morocco: Chefchaouen, path to Bab de Lars, Vargas, P. *152PV00 (MAUAM)* | KY798727 | KY798821 | KY798649 |
| (5) Morocco: Beni Snassen, Zegzel, Vargas, P. *156PV04 (MAUAM)* | KY798728 | KY798822 | KY798650 |
| (6) Morocco: Zegzel, Vargas, P. *194PV00 (MAUAM)* | KY798729 | KY798823 | KY798651 |
| (7) Morocco: Djebel Bouhalla, Valcárcel, V. *30VV03(1) (MAUAM)* | KY798730 | KY798824 | KY798652 |
| (8) Morocco: Azilal, cascadas de Ozoud, Vargas, P. *67PV05(2) (MAUAM)* | – | KY798825 | KY798653 |
| *Hedera nepalensis* K.Koch var. *nepalensis* |  |  |  |
| (1) India: Kashmir, Manat, McAllister, H.A. *246HAM (LIV)* | – | – | AF506102 |
| (2) India: Kashmir, Valcárcel, V. *03VV12 (MAUAM)* | KY798733 | KY798829 | KY798656 |
| (3) Nepal: Eastern Napal, Rolwaling, Khumbu, Chang, C.S. *s.n. (00061886SNUA)* | KY798731 | KY798826 | KY798654 |
| (4) Nepal: Tukache - Kokhethanti, Kali Gandaki river, *Moreno, J.C.* s.n. *(MAUAM)* | KY798732 | KY798827 | KY798655 |
| (5) Nepal: Lumle, Naga, Kasko, Pondet, R.C. *166 (028025WU)* | – | KY798828 | – |
| (6) Nepal: Sheopuri, north of Kathmandu, Chuma, Ch. *726584 (M03-01-15TI)* | – | KY798854 | – |
| (7) Nepal: Dhaulagiri Zone, Mustang Distr., Shuzuki, M. *et al. 88/40581 (M03-01-10TI)* | – | KY798855 | – |
| *Hedera nepalensis* K.Koch var. *sinensis* Rehder |  |  |  |
| (1) China: Xizang Province, Linzhi Xian, Dongjinxiang, Wen, J. *9172 (US)* | – | KY798835 | – |
| (2) China: Yunnan, Tengchong, Wen, J. *5682 (US)* | – | KY798836 | KY798660 |
| (3) China: Yunnan, Maguan, Wen, J. *5580 (US)* | – | KY798837 | KY798661 |
| (4) China: Yunnan, Tengchong, Wen, J. *5680 (US)* | KY798735 | KY798838 | KY798662 |
| (5) China: Yunnan, Baoshan, Wen, J. *6342 (US)* | KY798736 | KY798839 | KY798663 |
| (6) China: Yunnan, Gaoligongshan, Wen, J. *13862 (US)* | KY798738 | KY798841 | KY798665 |
| (7) China: Yunnan, Marlipo, Wen, J. *5627(10) (US)* | KY798739 | KY798842 | KY798666 |
| (8) China: Yunnan, Songming, Wen, J. *5745 (US)* | KY798753 | – | KY798674 |
| (9) China: SE Yunnan, Wen, J. *10634 (US)* | KY798737 | KY798840 | KY798664 |
| (10) China: SE Yunnan, *81150.2.2 (US)* | KY798740 | KY798843 | KY798667 |
| (11) China: SE Yunnan, Wen, J. *81097(212) (US)* | KY798752 | KY798845 | – |
| (12) China: SW Yunnan, Vargas, P. *206PV04 (MAUAM)* | KY798741 | KY798844 | KY798668 |
| (13) China: Sichuan, Leshon, Vargas, P. *119PV03 (MAUAM)* | KY798742 | KY798846 | KY798669 |
| (14) China: Sichuan, Vargas, P. *123PV07 (MAUAM)* | – | KY798847 | KY798670 |
| (15) China: Sichuan, Mt. Omei, Wen, J. *5013 (US)* | KY798743 | KY798848 | – |
| (16) China: Guangxi, Jingxiu Xian, Wen, J. *Wen11607(2) (US)* | KY798744 | KY798849 | KY798671 |
| (17) China: Zeilang, Chongging, Mt. Jinyun, Vargas, P. *120PV03(1) (MAUAM)* | KY798745 | KY798850 | KY798672 |
| (18) China: Zeilang, Lushan, Wen, J. *5523 (US)* | KY798754 | KY798851 | – |
| (19) China: Zeilang, Lushan, Taiyicum, Min-xiang, N. *92028 (TNM S18974)* | – | KY798852 | KY798673 |
| (20) China: Hubei, Lichuan, Wen, J. *8151 (US)* |  | KY798853 | – |
| (21) Vietnam: Fan-si Pan, McAllister, H.A. *895HAM (LIV)* | – | KY798830 | KY798657 |
| (22) Vietnam: Lao Cai Province, Sa Pa District, Sa Pa, Ham Rong Mountain, Wen, J. *5980 (US)* | KY798734 | KY798831 | KY798658 |
| (23) Vietnam: Lao Cai Province, Sa Pa District, Sa Pa, Ham Rong Mountain, Wen, J. *Wen10899 (US)* | – | KY798832 | KY798659 |
| (24) Vietnam: Lo Cai Province. Sa Pa District, Sa Xeng Village, Wen, J. *6083 (US)* | KY798751 | KY798833 | – |
| (25) Vietnam, *KR2884 (LIV)* | – | KY798834 | KY798675 |
| *Hedera pastuchowii* Woronow subsp. cypria (McAllister) Hand |  |  |  |
| (1) Cyprus: Limasol, Apsein, McAllister, H.A. *504HAM (LIV)* | – | – | AF506113 |
| (2) Cyprus: Troodos Mountains, Kakopetria, McAllister, H.A. *188HAM (LIV)* | – | – | AF506095 |
| (3) Cyprus: Prodromus - Kakopetria, Valcárcel, V. *07VV05(4) (MAUAM)* | KY798692 | KY798770 | KY798599 |
| (4) Cyprus: Platres, Valcárcel, V. *12VV05(11) (MAUAM)* | KY798693 | KY798771 | KY798600 |
| *Hedera pastuchowii* Woronow subsp. *pastuchowii* |  |  |  |
| (1) Afghanistan: Kunar, Dewagal Darrah, Umgebung von Chalas, Anders, O. *11052 (MSB01495)* | KY798755 | – | - |
| (2) Caucasus: Cultivated in Edinburgh from Caucasus material, *(MAUAM)* | KY798746 | KY798856 | KY798676 |
| (3) Iran: Elburz mountains, McAllister, H.A. *259HAM (MA)* | – | – | AF506103 |
| (4) Iran: Mazanderan, Talair valley, Aramshad, Zumer, M. *74bMZ (BG)* | – | KY798857 | – |
| (5) Iran: Haraz, Valley Kareshang, Wendelbo, P. *s.n. (1950BG)* | – | KY798858 | – |
| *Hedera rhombea* (Miq.) Bean |  |  |  |
| (1) Japan: Shizuoka, Fukuroi-shi, Mt. Ogasayama, Tadashi, Y. *3765YT (TAIPEI 096390TAIF)* | – | KY798866 | KY798682 |
| (2) Japan: Shizouka Pref., Kamo-gun, Higashi izu-cho, along the Kawakubo-kawa, Murata, J. *et al. s.n. (M03-01-57TI)* | – | KY798868 | – |
| (3) Japan: Natou Hsien, Jenai Hsiang, Junghsing Village, Yang, T.Y.A. *et al.* *11706 (TNM S70029)* | KY798747 | KY798859 | JQ745330 |
| (4) Japan: Ishikawa Pref. *Fujii 7390F (5290MAKINO)* | – | – | JQ745331 |
| (5) Japan: Tokyo, Mt Takanose, *K. Suzuki 9676 (270910MAKINO)* | – | – | JQ745325 |
| (6) Japan: Oita, usa-jingir, Esténabez, B. *s.n. (MAUAM)* | – | KY798860 | KY798677 |
| (7) Korea: South coast, *S. Lee s.n. (LIV)* | – | – | AF506099 |
| (8) Korea: Ulluengdo Island, Baek, W.K. *s.n. (0013651GANGWON)* | KY798757 | KY798867 | – |
| (9) Taiwan: Chiayi, Sheau Tashan, Hwang, Y.H. & Chen, S.J. *s.n. (TNM S9202)* | – | – | KY798681 |
| (10) Taiwan: Taipei Tatunshan, *Chian 3308 (180551TAIF)* | – | – | JQ745323 |
| (11) Taiwan: Taipei, Wang, C.M. & Tsai, Y.H. *03011 (TNM S48777)* | – | – | JQ745324 |
| (12) Taiwan: Taichung, *Lii 348 (152957TAIF)* | – | – | JQ745326 |
| (13) Taiwan: Nagano, Asano, K & Asano, H. *s.n. (336811MAKINO)* | – | – | JQ745327 |
| (14) Taiwan: Taoyuan, *Kuo, S.M. 206 (178186TAIPEI)* | – | – | JQ745328 |
| (15) Taiwan: Taipei, Tansmingshan National Park, McAllister, H.A. *869HAM (LIV)* | KY798748 | KY798861 | KY798678 |
| (16) Taiwan: Taichung Hsien, Hoping Hsiang, Shen, H.Y. *et al. 415 (TNM S16067)* | – | KY798862 | KY798679 |
| (17) Taiwan, Liu *s.n. (US)* | – | KY798863 | KY798680 |
| (18) Taiwan: Miaoli Co., Wu Feng Hsiang, Kuanwu, Chen, C.H. *et al. 824 (TAIPEI 075074)* | – | KY798864 | JQ745332 |
| (19) Taiwan: Ilan County, Ssuyuan, Cheng *3856 (TAIPEI 139079TAIF)* | KY798756 | KY798865 | – |
