# Supplementary material for: Phylogenetic and paleobotanical evidence for late Miocene diversification of the Tertiary subtropical lineage of ivies (Hedera L., Araliaceae)
Source: BMC Evol Biol. 2017 Jun 22;17:146. doi: 10.1186/s12862-017-0984-1 (PMC5480257; doi:10.1186/s12862-017-0984-1)
Supplement: Supplementary file 3 — Summary of DNA sequences variation and evolutionary models best fitting the nuclear and plastid matrices. (DOCX 41 kb) [file 12862_2017_984_MOESM3_ESM.docx]

|  | | | **nrITS** | ***trn*H-*psb*A** | ***trn*T- *trn*L** | ***rpl*32** |
| --- | --- | --- | --- | --- | --- | --- |
| **Evolutionary model selected** | | GTR+I+G (ITS1, ITS2)  JC (5.8S) | F81 | GTR+G | F81 |  |
| **Number of taxa** | | 90 | 112 | 134 | 77 |  |
| Total length | 598 | 422 | 808 | 759 |  |  |
| **Complete dataset** | |  |  |  |  |  |
| Variable characters | 260 | 8 | 35 | 22 |  |  |
| Informative characters | 170 | 5 | 8 | 6 |  |  |
| **Hedera** | |  |  |  |  |  |
| Variable characters | 37 | 6 | 29 | 13 |  |  |
| Informative characters | 28 | 5 | 8 | 6 |  |  |
