# Supplementary figures and images for: Phylogenetic and paleobotanical evidence for late Miocene diversification of the Tertiary subtropical lineage of ivies (Hedera L., Araliaceae)
Source: BMC Evol Biol. 2017 Jun 22;17:146. doi: 10.1186/s12862-017-0984-1 (PMC5480257; doi:10.1186/s12862-017-0984-1)

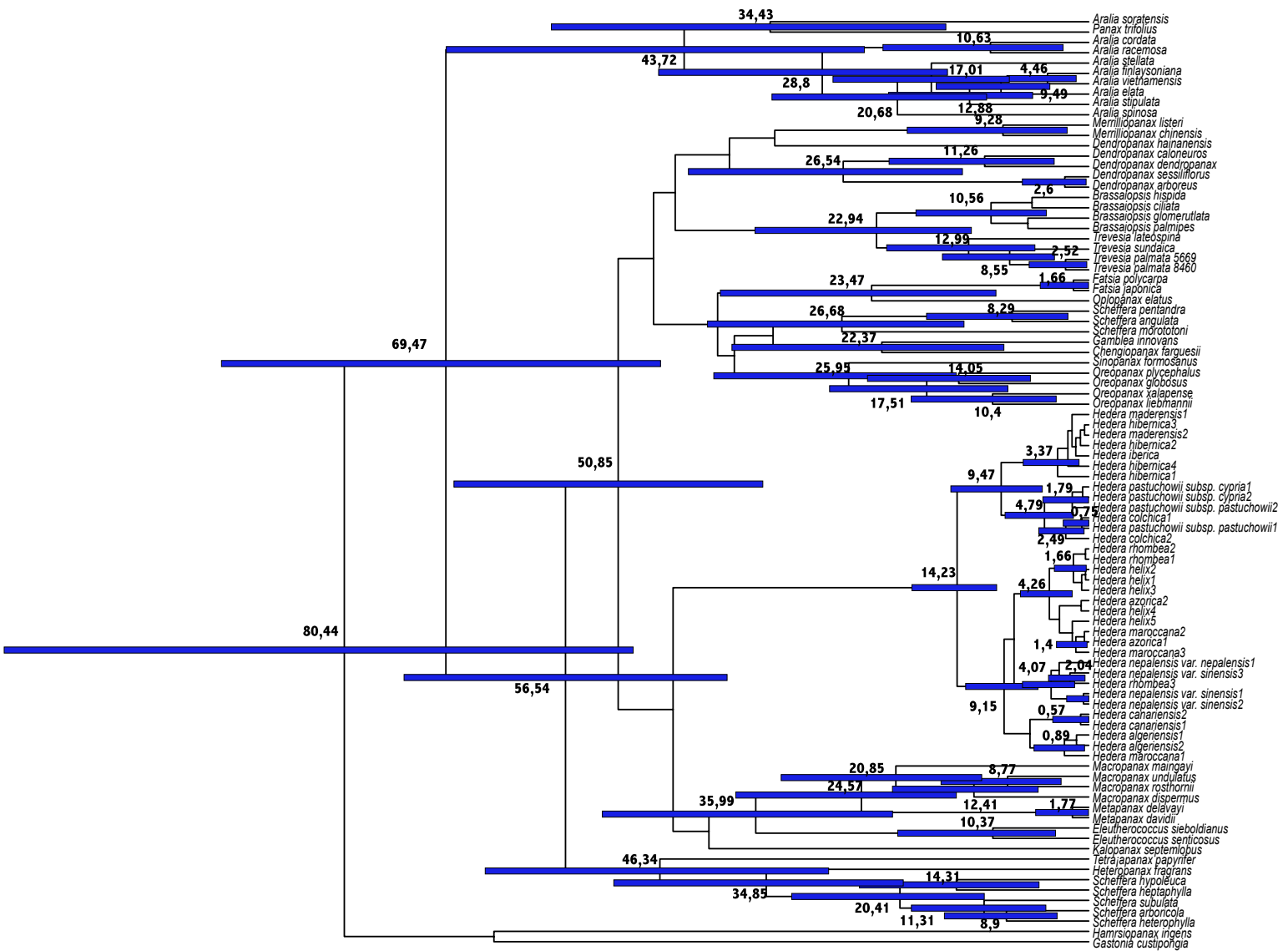

Supplement: Supplementary file 5 — Beast Maximum Clade Credibility chronogram of the nrITS dataset of Araliaceae with the secondary calibration approach. Legend: Mean ages and 95% CI are only represented for clades with >0.5 Posterior Probability support. (PDF 255 kb) [file 12862_2017_984_MOESM5_ESM.pdf]

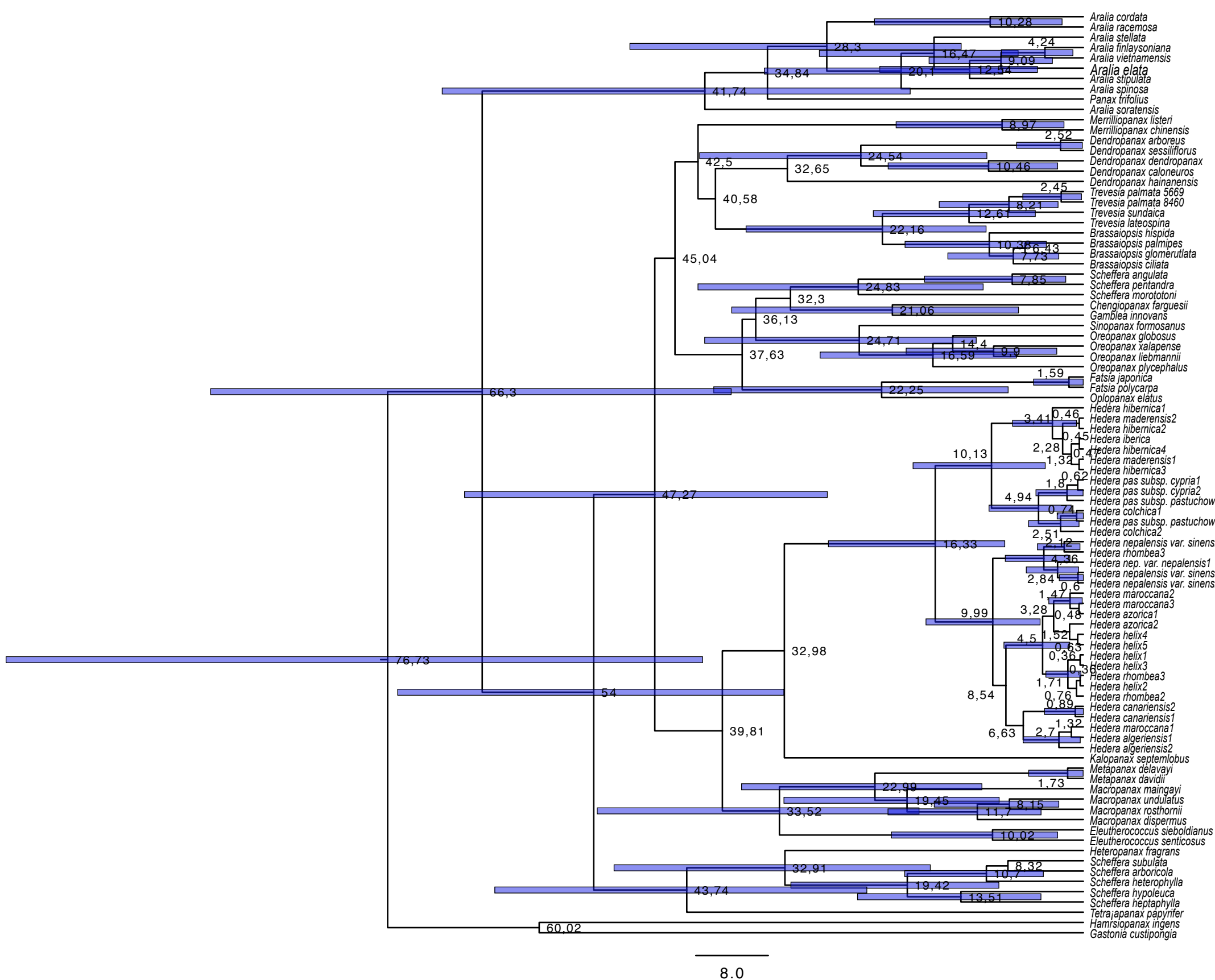

Supplement: Supplementary file 6 — Beast Maximum Clade Credibility chronogram of the nrITS dataset of Araliaceae with the fossil calibration approach. Mean ages and 95% CI are only represented for clades with >0.5 Posterior Probability support. (PDF 236 kb) [file 12862_2017_984_MOESM6_ESM.pdf]

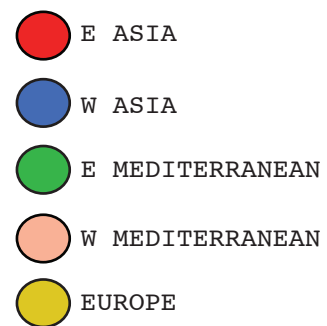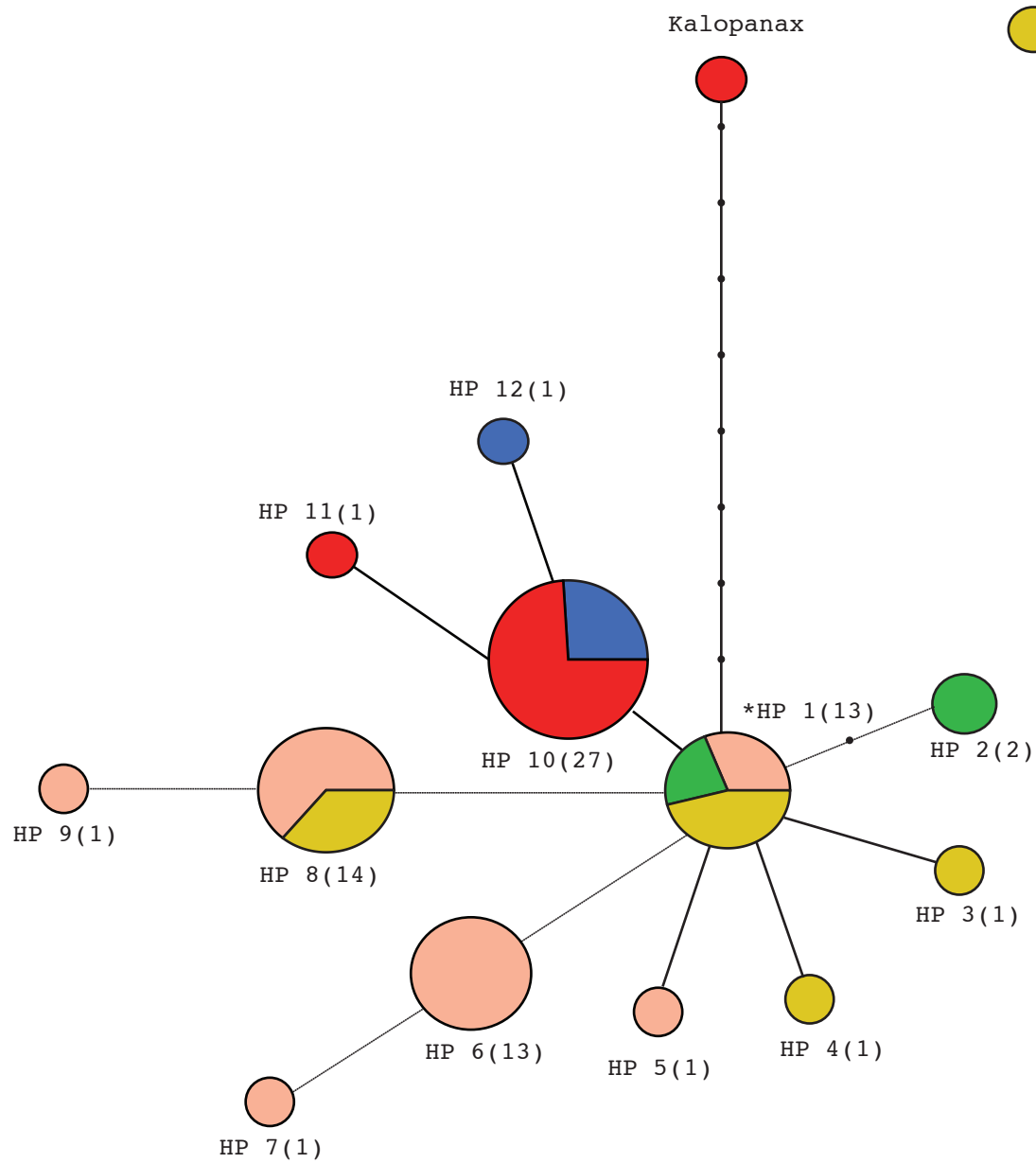

Supplement: Supplementary file 9 — Hedera phylogeographic network obtained from the Statistical Parsimony analysis of the rpL32 plastid region. Haplotype numbering is according to Additional file 8. Circle dimensions are proportional to the number of samples displaying each haplotype. Kalopanax septembolus is used as outgroup. (PDF 96 kb) [file 12862_2017_984_MOESM9_ESM.pdf]

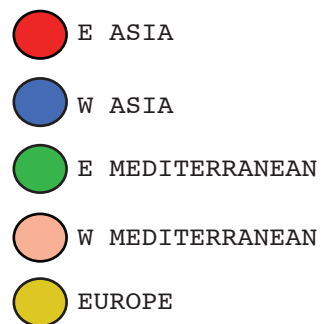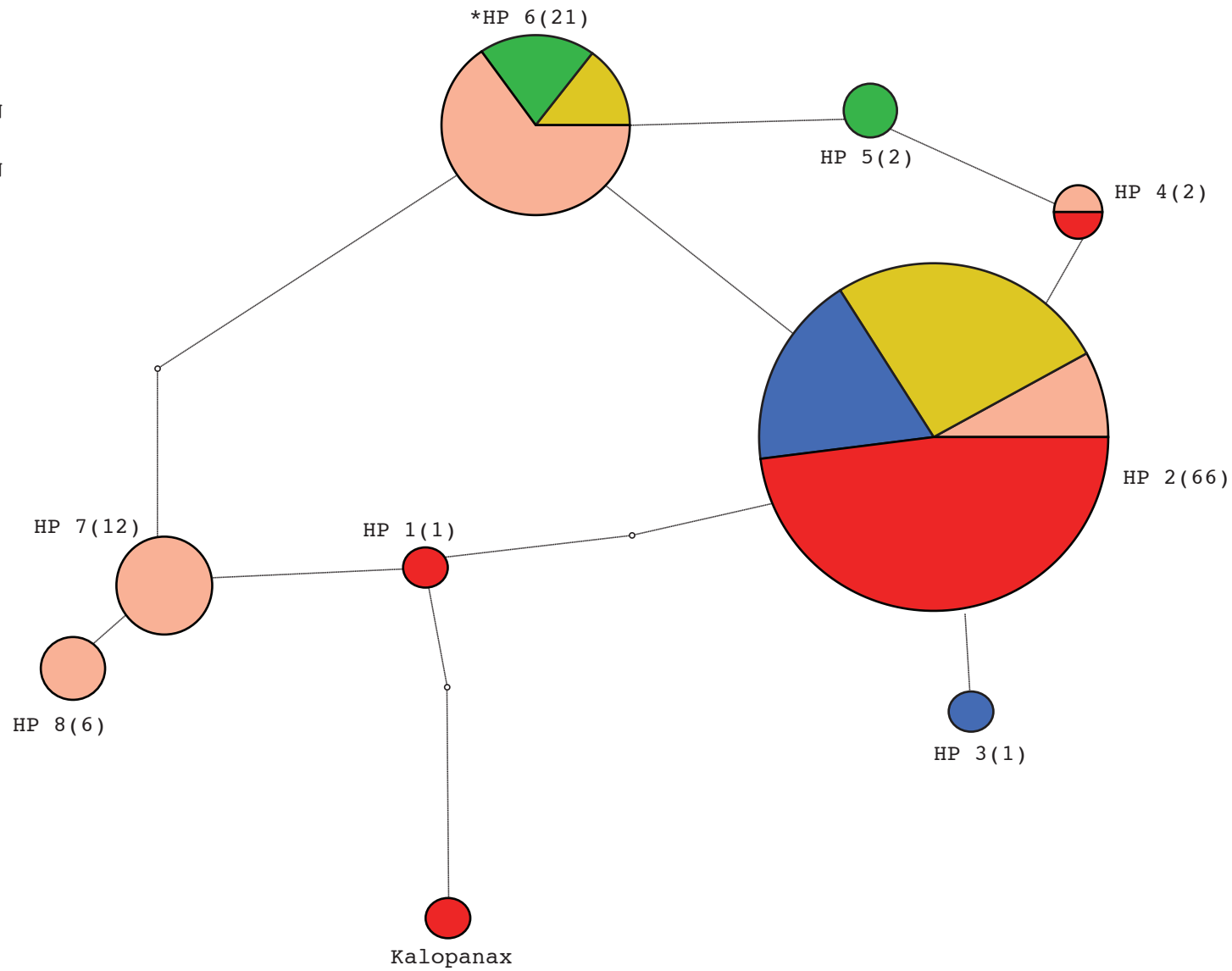

Supplement: Supplementary file 10 — Hedera phylogeographic network obtained from the Statistical Parsimony analysis of the trnH-psbA plastid region. Haplotype numbering is according to Additional file 8. Circle dimensions are proportional to the number of samples displaying each haplotype. Kalopanax septembolus is used as outgroup. (PDF 93 kb) [file 12862_2017_984_MOESM10_ESM.pdf]

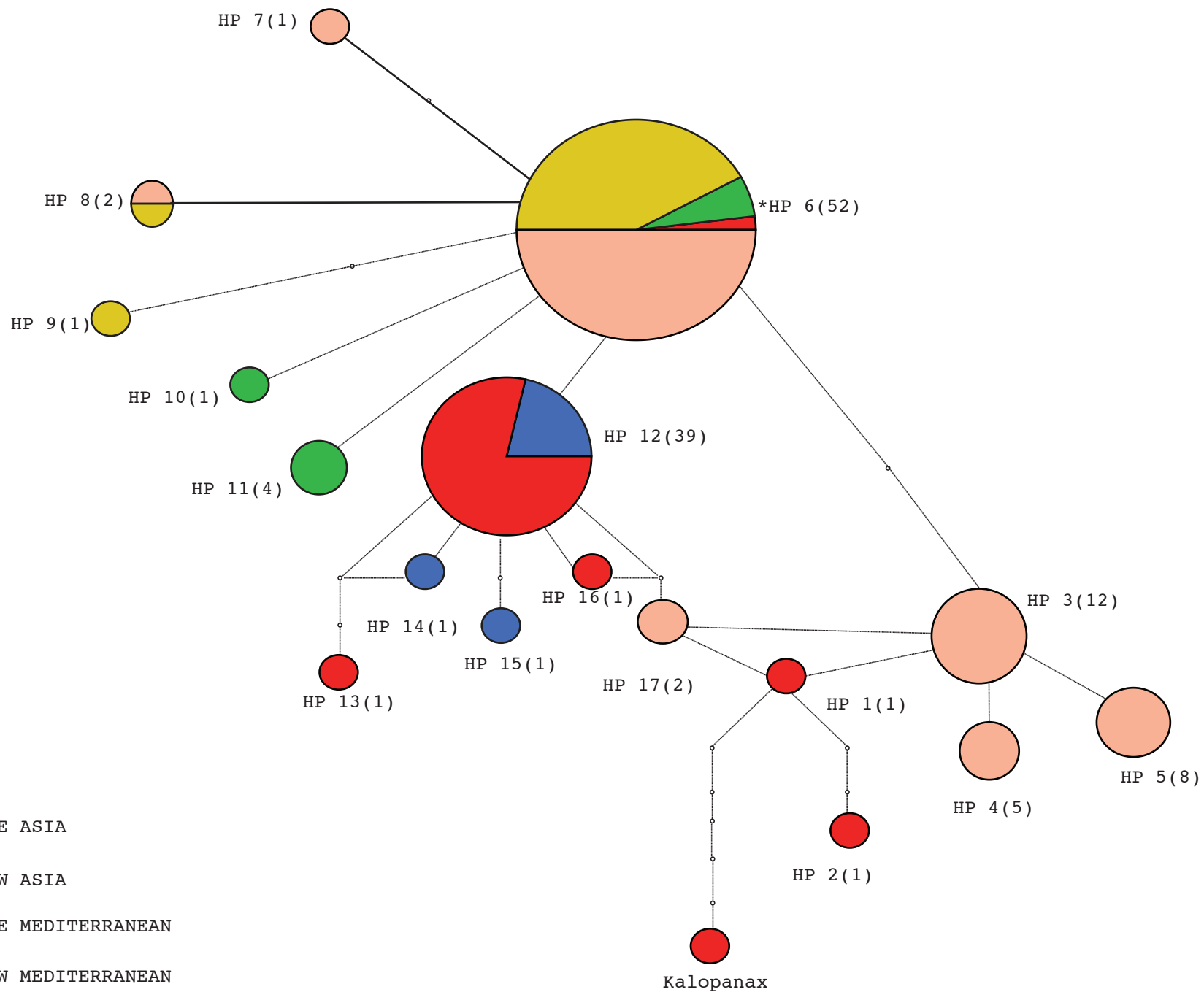

Supplement: Supplementary file 11 — Hedera phylogeographic network obtained from the Statistical Parsimony analysis of the trnT-trnL plastid region. Haplotype numbering is according to Additional file 8. Circle dimensions are proportional to the number of samples displaying each haplotype. Kalopanax septembolus is used as outgroup. (PDF 108 kb) [file 12862_2017_984_MOESM11_ESM.pdf]

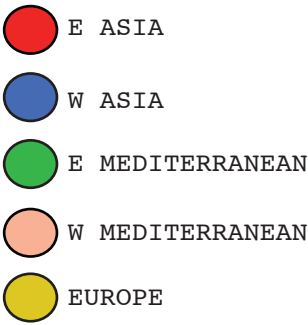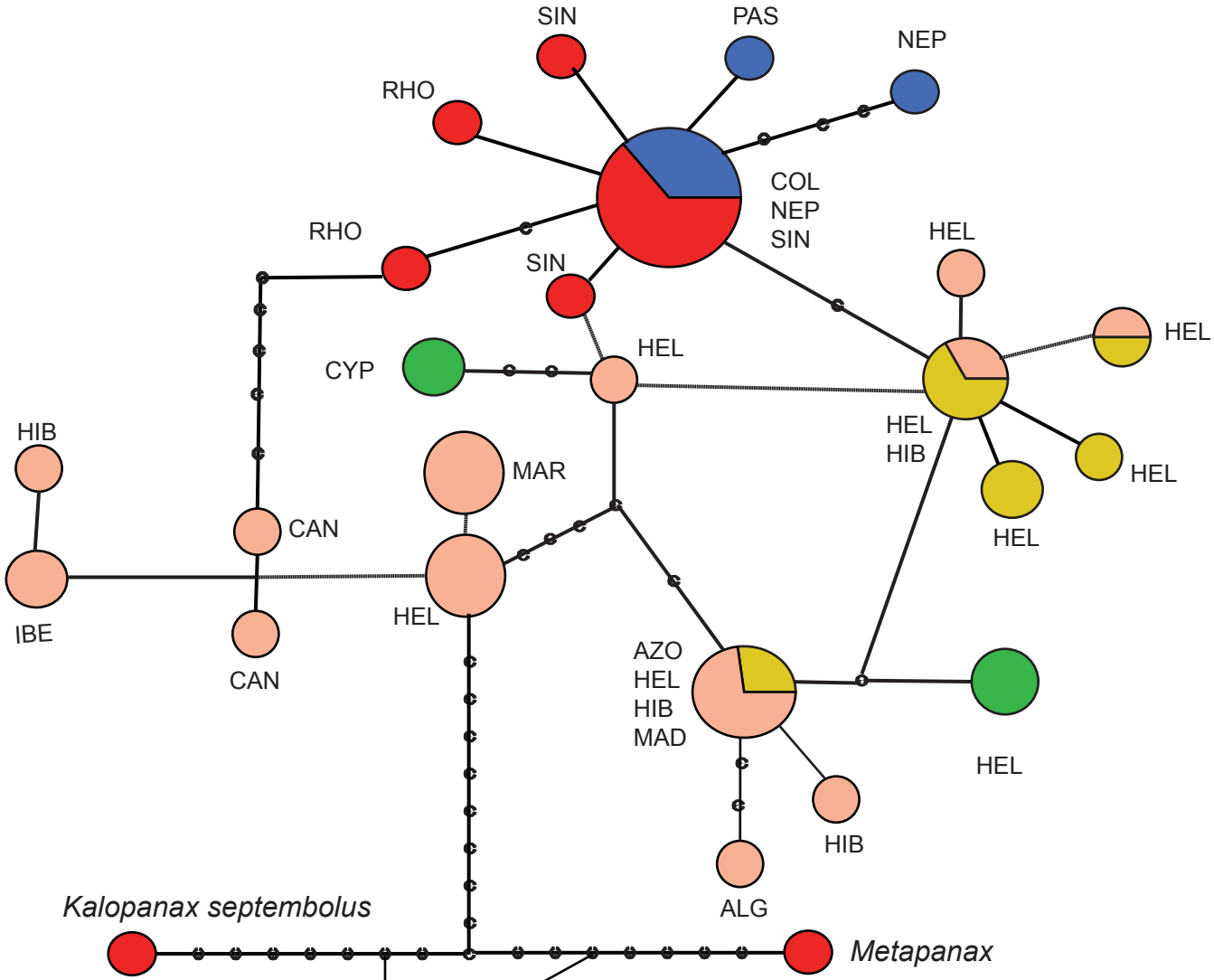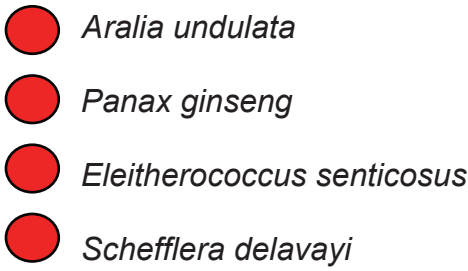

*Brassaiopsis hainla*

Supplement: Supplementary file 12 — Hedera phylogeographic network obtained from the Statistical Parsimony analysis of the three-plastid regions (trnH-psbA, trnT-trnL, rpL32) and using seven Araliaceae genera as outgroup. Circle dimensions are proportional to the number of samples displaying each haplotype. Abbreviation names of the taxa displaying each haplotype are as follows: ALG, H. algeriensis; AZO, H. azorica; CAN, H. canariensis; COL, H. colchica; CYP, H. pastuchowii subsp. cypria; HEL, H. helix; HIB, H. hibernica; IBE, H. iberica; MAD, H. maderensis; MAR, H. maroccana; NEP, H. nepalensis var. nepalensis; PAS, H. pastuchowii subsp. pastuchowii; RHO, H. rhombea; SIN, H. nepalensis var. sinensis. (PDF 188 kb) [file 12862_2017_984_MOESM12_ESM.pdf]
