# Supplementary material for: Phylogenetic and paleobotanical evidence for late Miocene diversification of the Tertiary subtropical lineage of ivies (Hedera L., Araliaceae)
Source: BMC Evol Biol. 2017 Jun 22;17:146. doi: 10.1186/s12862-017-0984-1 (PMC5480257; doi:10.1186/s12862-017-0984-1)
Supplement: Supplementary file 7 — Fitness to speciation models. Summary of the results of the fitness to speciation models estimated from 6500 posterior pruned trees of the Hedera clade. N indicates the number of trees that recover a given evolutionary model. Mean values and 95% CI are provided for AIC and each of the parameters of the models. (DOCX 49 kb) [file 12862_2017_984_MOESM7_ESM.docx]

| model | N | AIC | a | r1 | r2 | r3 | st1 | st3 |
| --- | --- | --- | --- | --- | --- | --- | --- | --- |
| bd | 2495 | 16.41  (11.85-20.97) | 0.99 | 0.002 | - | - | - | - |
| pureBirth | 2 | 16.91-23.23 | - | 0.14-0.25 | - | - | - | - |
| yule2rate | 1958 | 12.16  (6.86-17.46) | - | 0.04  (-0.14-0.23) | 0.29  (0.024-0.55) | - | 7.82  (5.94-9.71) | - |
| Yule3rate | 2045 | 12.51  (7.31-17.7) | - | 0.03  (-0.27-0.34) | 0.94  (-2.61-4.45) | 0.36  (-1.11-1.84) | 10.19  (7.42-12.97) | 3.20  (0.35-6.05) |
