# Supplementary material for: Phylogenetic and paleobotanical evidence for late Miocene diversification of the Tertiary subtropical lineage of ivies (Hedera L., Araliaceae)
Source: BMC Evol Biol. 2017 Jun 22;17:146. doi: 10.1186/s12862-017-0984-1 (PMC5480257; doi:10.1186/s12862-017-0984-1)
Supplement: Supplementary file 8 — List of the haplotypes detected in the samples included in the phylogeographic study. Taxa name and general distribution is provided. Locality and voucher is specified for each sample as well as the number of haplotype detected individually for the three regions (rpL32, trnH-psbA, trnT-trnL). Last column indicates the number of haplotype when combining the three-plastid regions rpL32, trnH-psbA and trnT-trnL (HP3). (DOCX 106 kb) [file 12862_2017_984_MOESM8_ESM.docx]

|  | | ***HP^rpL32^*** | ***HP^trnH-psbA^*** | ***HP^trnT-L^*** | ***HP^3^*** |
| --- | --- | --- | --- | --- | --- |
| ***Kalopanax septemlobus*** (Thunb.) Koidz. |  | | | | |
| (1) China: *R. Li 552* (KUN) | | Kalopanax | Kalopanax | Kalopanax | Kalopanax |
| ***Hedera algeriensis Hibberd*** | **North Africa: Algeria and Tunisia** | | | | |
| (1) Algeria: Kabylie mountains, *McAllister, H.A. 838HAM (LIV)* | | – | 6 | 12 | – |
| (2) Tunisia: Ain-Draham, *Aldasoro, J.J.* *A2890(5) (MA)* | | 8 | 6 | 7 | 24 |
| ***Hedera azorica*** (Gand) Carrière | **Portugal: Azores Islands** | | | | |
| (1) Pico, *McAllister, H.A.* *279HAM (LIV)* | | – | – | 6 | – |
| (2) Pico, Cabeco das Cabras, *Martínez, J.* *216JM04 (MAUAM)* | | 8 | 6 | 6 | 22 |
| (3) Sao Miguel, *McAllister, H.A.* *947HAM (LIV)* | | – | – | 6 | – |
| (4) Sao Jorge, Faja, *Martínez, J.* *225JM04 (MAUAM)* | | 8 | 6 | 6 | 22 |
| (5) Faial, Caldeira (Horta), *Martínez, J.* *212JM04 (MAUAM)* | | 8 | 6 | 6 | 22 |
| ***Hedera canariensis*** Willd. | **Spain: Canary Islands** | | | | |
| (1) Gran Canaria, Valleseco, *Vasák, V.* *s.n. (BR-SP852826)* | | – | 7 | – | – |
| (2) Tenerife, Chinobre, Las Mercedes, *Martínez, J.* *237HAM (LIV)* | | – | – | 3 | – |
| (3) La Gomera, Parque Natural de Garajonay, *Valcárcel, V. 58VV00 (MAUAM)* | | – | – | 17 | – |
| (4) La Gomera, P. N. Garajonay, El Cedro, *Vargas, P.* *172PV05 (MAUAM)* | | 6 | 8 | 3 | 3 |
| (5) La Palma, La Galga, el Cubo de la Galga, *Valcárcel, V.* *66VV04(11) (MAUAM)* | | 6 | 8 | 17 | 6 |
| ***Hedera colchica*** (K.Koch) K.Koch | **North and South Caucasus and Turkey** | | | | |
| (1) Georgia: T'elavi, Zagodeki, *McAllister, H.A.* *470HAM (LIV)* | | – | – | 12 | – |
| (2) Georgia: Lagodekhi, *Opred, I.* *s.n. M0080097* | | 10 | 2 | 12 | 8 |
| (3) Georgia: Prov. Krasnodar, between Babuk-Aul and Solokh-Aul, *Latschaschvili, J. s.n. (576348MA)* | | – | 2 | – | – |
| (4) Russia: Krasnodar, Krashodarsky Krai, *Wen, J.* *10358 (US)* | | 10 | 2 | 12 | 8 |
| (5) Turkey: Rize, near Ikizdere, *Nisa, S. 760SN (MA689076)* | | 10 | 2 | 12 | 8 |
| ***Hedera helix*** L. | **Europe, Caucasus and Turkey** | | | | |
| (1) Austria: Baden, *Valcárcel, V.* *42VV03(2) (MAUAM)* | | – | 2 | 6 | – |
| (2) Belgium: Brussels, Gossellies, *Vargas, P.* *114PV03(1) (MAUAM)* | | 3 | 2 | 6 | 18 |
| (3) Denmark: Jernhatten, Mols, *Larsen, K.* *275 (MA186341)* | | – | – | 6 | – |
| (4) France: Corsica, *Valcárcel, V.* *78VV00 (MAUAM)* | | 1 | 2 | 8 | 19 |
| (5) France: Chizé, *Grivet, D. 12HH7 (MAUAM)* | | 1 | – | 6 | – |
| (6) France: Moint Ventoux, near Malaucena, *Valcárcel, V.* *155VV01 (MAUAM)* | | – | 2 | 6 | – |
| (7) France: Pyrenees, Gabas, Portalet pass, *Vargas, P.* *338PV02(3) (MAUAM)* | | 1 | 2 | 6 | 16 |
| (8) France: Fontainebleau, *Grivet, D.* *11HH10 (MAUAM)* | | – | 2 | – | – |
| (9) Germany: Wiesbaden, Eberbach Kloster, *Vargas, P.* *169PV01(1) (MAUAM)* | | 1 | 2 | 8 | 19 |
| (10) Germany: Bad Reichen hall, Sch. Weissbach, *Vargas, P.* *386PV02(2) (MAUAM)* | | – | 2 | 6 | – |
| (11) Greece: Crete, between Vlatos and Ellos, Strovles, *Vargas, P.* *116PV05 (MA)* | | 1 | 6 | 11 | 21 |
| (12) Greece: Peloponeso, A. Kalovryta, *Vargas, P. 395PV02(1)bis (MAUAM)* | | 1 | 6 | 11 | 21 |
| (13) Hungary: Budapest, Pest, *Vargas, P.* *106PV03(2) (MAUAM)* | | – | 2 | 6 | – |
| (14) Italy: Ercolano, Vesubio, *Vargas, P.* *215PV01(2) (MAUAM)* | | 5 | 2 | 6 | 17 |
| (15) Italy: Godi pass, Scanno, *Vargas, P.* *217PV01(1) (MAUAM)* | | – | – | 6 | – |
| (16) Italy: Sicily, Siracusa, Sortino, Iblei mountain, *Herrero, A.* *AH1002 (MA)* | | 1 | 4 | 6 | 14 |
| (17) Moravia: Moravia septenrionalis, distr. Sumperk, in vicinitate pagi Temenice, loco "Pod Lvákem" dicto., *Vasák, V.* *s.n. (918956BR)* | | – | 2 | 6 | – |
| (18) Poland: Pogórze Wisnickie foothills, vicinity of Gosprzydowa, *Vargas, P.* *117PV03(1) (MAUAM)* | | 4 | 2 | 6 | 20 |
| (19) Spain: Huesca, *Vargas, P. s.n. (MA)* | | – | – | 9 | – |
| (20) Spain: Huesca, Linas de Broto, near Cotefablo Pass, *Vargas, P.* *335PV02(2) (MAUAM)* | | 8 | – | 6 | – |
| (21) Spain: Almería, Sierra de Gádor, Fondón, *Vargas, P.* *14PV05(4) (MAUAM)* | | – | 7 | 3 | – |
| (22) Spain: Gerona, *Nieto Feliner, G.* *4344GN (MA)* | | – | – | 6 | – |
| (23) Spain: Guadalajara, Tamajón, Retiendas, *Valcárcel, V.* *4VV01 (MAUAM)* | | – | 7 | 3 | – |
| (24) Spain: Granada, near Competa, Salto de Maroma, *McAllister, H.A.* *953HAM (LIV)* | | – | – | 3 | – |
| (25) Spain: Granada, Sierra Nevada, Alpujarras, Maza de Lino, *Vargas, P.* *121PV04 (MAUAM)* | | 6 | 7 | 3 | 1 |
| (26) Spain: Jaén, Cazorla, *Valcárcel, V.* *16VV02 (MAUAM)* | | 6 | 7 | 3 | 1 |
| (27) Spain: Madrid, Valle del Paular, *Vargas, P.* *387PV02(8) (MAUAM)* | | 6 | 7 | 3 | 1 |
| (28) Spain: Málaga, *McAllister, H.A. s.n. (LIV)* | | – | – | 5 | – |
| (29) Spain: Málaga, Ronda, El Quejigal, *Vargas, P.* *5PV97 (MAUAM)* | | – | – | 4 | – |
| (30) Spain: Menorca, *Valcárcel, V.* *37VV03(1) (MAUAM)* | | 1 | 2 | 6 | 16 |
| (31) Spain: Murcia, *Aedo, C.* *5934CA (MA)* | | – | – | 5 | – |
| (32) Spain: Soria, *Valcárcel, V.* *9VV01 (MAUAM)* | | 1 | 2 | 6 | 16 |
| (33) Spain: Valencia, Castellón, Bellavista, *Guzmán, B. 127BGA04(2)* | | 8 | 6 | 6 | 22 |
| (34) Switzerland: Murten, *Valcárcel, V.* *17VV01 (MAUAM)* | | 8 | 2 | 6 | 18 |
| (35) Turkey: Mugla, *Compton, J.A.* *s.n. (LIV)* | | – | – | 11 | – |
| (36) Turkey: Zonguldak, Ahmetusta pass, *Aedo, C.* *6519CA (MA)* | | – | 6 | – | – |
| (37) United Kingdom: Scotland, S.Uist, S.Glendale, Bagh Mor., *McAllister, H.A.* *570HAM (LIV)* | | – | – | 6 | – |
| (38) United Kingdom: Scotland, South west Hebrides, *Valcárcel, V.* *430VV01 (MAUAM)* | | – | 2 | 6 | – |
| (39) Ukraine: Crimea, near Yalta, Uchan- Su waterfall, *Vargas, P.* *116PV03 (MAUAM)* | | 1 | 6 | 11 | 21 |
| (40) Ukraine: Crimea, Yalta, *Valcárcel, V.* *427VV01 (MAUAM)* | | – | 6 | – | – |
| ***Hedera hibernica*** (G.Kirchn.) Bean | **Atlantic Europe: United Kingdom, France, Portugal and Spain** | | | | |
| (1) Portugal: Lindoso, *McAllister, H.A.* *925HAM (LIV)* | | – | – | 6 | – |
| (2) Portugal: Louso, Serra do Caramulo, *Ribeiro, P.* *336PR (MAUAM)* | | 8 | 6 | 6 | 22 |
| (3) France: St Chinian, *Vargas, P.* *229PV06 (MAUAM)* | | 1 | 2 | 6 | 16 |
| (4) France: St Andrea Di bozio, *Grivet, D.* *15HH02 (MAUAM)* | | 9 | 6 | 6 | 23 |
| (5) Spain: Asturias, Monte Andorso, Valdés, *McAllister, H.A.* *937HAM (LIV)* | | – | – | 6 | – |
| (6) Spain: Cádiz, Grazalema, Las Palomas pass, *Valcárcel, V.* *103VV00 (MAUAM)* | | 7 | 8 | 4 | 5 |
| (7) Spain: Granada, near Competa, *McAllister, H.A.* *949HAM (LIV)* | | – | – | 3 | – |
| (8) Spain: Gerona, *Nieto Feliner, G.* *4615GN(3) (MA)* | | 8 | 6 | 6 | 22 |
| (9) Spain: Huelva, Jabugo, *McAllister, H.A.* *545HAM (LIV)* | | – | – | 6 | – |
| (10) Spain: Málaga, between Antequera and Ayora, *Valcárcel, V.* *8VV02(1) (MAUAM)* | | 6 | 7 | 3 | 1 |
| (11) Spain: Santander, Bollacín, El Escudo pass, *Vargas, P.* *127PV01(6) (MAUAM)* | | 8 | 6 | 6 | 22 |
| (12) United Kingdom: Ireland, Cashel  Vargas, P. *180PV10(1) (MAUAM)* | | 1 | 2 | 6 | 16 |
| (13) United Kingdom: Ireland, Torc Waterfall, *Vargas, P.* *171PV10(1) (MAUAM)* | | 1 | 2 | 6 | 16 |
| (14) United Kingdom: Ireland, Glengarriff, *Vargas, P.* *177PV10(2) (MAUAM)* | | 8 | 6 | 6 | 22 |
| (15) United Kingdom: Ireland, Crookstown, *Vargas, P.* *170PV10 (MAUAM)* | | 8 | 6 | 6 | 22 |
| (16) United Kingdom: Scotland, Drumnadrochit, *Vargas, P.* *70PV12(1) (MAUAM)* | | – | 2 | 6 | – |
| (17) United Kingdom: Scotland, Island of Skye, Portree, *Vargas, P.* *73PV12 (MAUAM)* | | – | 2 | 6 | – |
| (18) United Kingdom: Scotland, Edinburgh, *Vargas, P.* *75PV12 (MAUAM)* | | – | 2 | 6 | – |
| ***Hedera iberica*** (McAllister) Ackerfield & J.Wen | **Southwestern Europe: S Portugal and SW Spain** | | | | |
| (1) Spain: Cádiz, Los Barrios, *McAllister, H.A.* *15HAM (LIV)* | | – | – | 3 | – |
| (2) Spain: Cádiz, Alcalá de los Gazules, "Porto Oscuro", *Valcárcel, V.* *392VV01(1) (MAUAM)* | | 6 | 8 | 4 | 4 |
| (3) Spain: Cádiz, Algeciras-Tarifa, "El Bujeo", Sierra de Luna, *Valcárcel, V.* *399VV01(2) (MAUAM)* | | – | 8 | 4 | – |
| (4) Spain: Málaga, Sierra Bermeja, *Valcárcel, V.* *400VV01bis (MAUAM)* | | 6 | 8 | 4 | 4 |
| (5) Portugal: Monchique Mountains, *Segura Zubizarreta, A. 2017 (MA350593)* | | – | – | 3 | – |
| ***Hedera maderensis*** K.Koch ex A.Rutherf. | **Portugal: Madeira** | | | | |
| (1) Funchal, *McAllister, H.A.* *18HAM (LIV)* | | – | – | 6 | – |
| (2) Das Queimadas Park, *Franquinho L.O.* *s.n. (LIV)* | | – | – | 6 | – |
| (3) Santa near Achada da Cruz, *Valcárcel, V.* *05VV08(10) (MAUAM)* | | – | 6 | 6 | – |
| (4) Funchal, from Achadas da Cruz to Moniz pass, *Vargas, P.* 325PV00 (MA654987) | | 8 | 6 | 6 | 22 |
| (5) Levada de Casa do Lombo do Mouro, Navarro, C. *CN3395 (MA654727)* | | 8 | 6 | 6 | 22 |
| ***Hedera maroccana*** McAllister | **North Africa: Morocco** | | | | |
| (1) Marrakech, 31 Km south from Ourika valley, *McAllister, H.A.* *861HAM (LIV)* | | – | 6 | – | – |
| (2) Tetuan, Rift, Idit 10 Km east from Bou Azzer, *McAllister, H.A.* *868HAM (LIV)* | | – | – | 5 | – |
| (3) Gurugu Mountain, *Vargas, P.* *137PV04(7) (MAUAM)* | | 6 | 7 | 5 | 2 |
| (4) Chefchaouen, path to Bab de Lars, *Vargas, P.* *152PV00 (MAUAM)* | | 6 | 7 | 5 | 2 |
| (5) Beni Snassen, Zegzel, *Vargas, P.* *156PV04 (MAUAM)* | | 6 | 7 | 5 | 2 |
| (6) Zegzel, *Vargas, P.* *194PV00 (MAUAM)* | | 6 | 7 | 5 | 2 |
| (7) Djebel Bouhalla, *Valcárcel, V.* *30VV03(1) (MAUAM)* | | 6 | 7 | 5 | 2 |
| (8) Azilal, cascadas de Ozoud, *Vargas, P.* *67PV05(2) (MAUAM)* | | – | 6 | 6 | – |
| ***Hedera nepalensis*** K.Koch var. ***nepalensis*** | **W Continental Asia: from Pakistan to Nepal and India** | | | | |
| (1) India: Kashmir, Manat, *McAllister, H.A.* *246HAM (LIV)* | | – | – | 12 | – |
| (2) India: Kashmir, *Valcárcel, V.* *03VV12 (MAUAM)* | | 10 | 2 | 12 | 8 |
| (3) Nepal: E Nepal, Rolwaling, Khumbu, *Chang, C.S. s.n. (00061886SNUA)* | | 10 | 2 | 12 | 8 |
| (4) Nepal: C Nepal, Lumle, Naga, Kasko, Pondet, R.C. *166 (028025WU)* | | – | 2 | – | – |
| (5) Nepal: C Nepal, Sheopuri, north of Kathmandu, Chuma, Ch. *726584 (M03-01-15TI)* | | – | 2 | – | – |
| (6) Nepal: C Nepal, Dhaulagiri Zone, Mustang Distr., *Shuzuki, M.* *et al. 88/40581 (M03-01-10TI)* | | – | 2 | – | – |
| (7) Nepal: W Nepal, Tukache - Kokhethanti, Kali Gandaki river, *Moreno, J.C.* s.n. *(MAUAM)* | | 12 | 3 | 15 | 10 |
| ***Hedera nepalensis*** K.Koch var. ***sinensis*** Rehder | **E Continental Asia: from Vietnam and SW China to E China** | | | | |
| (1) China: Hubei, Lichuan, Wen, J. *8151 (US)* | | 10 | 2 | – | – |
| (2) China: Guangxi, Jingxiu Xian, *Wen, J*. *11607(2) (US)* | | 10 | 4 | 12 | 13 |
| (3) China: Sichuan, Leshan, *Vargas, P.* *119PV03* | | 10 | 2 | 12 | 8 |
| (4) China: Sichuan, *Vargas, P.* *123PV07* | | – | 2 | 12 | – |
| (5) China: Sichuan, Mt. Omei, *Wen, J. 5013 (US)* | | 10 | 2 | – | – |
| (6) China: Yunnan, Songming, *Wen, J.* *5745 (US)* | | 10 | – | 12 | – |
| (7) China: Yunnan, Tengchong, *Wen, J.* *5682 (US)* | | – | 2 | 12 | – |
| (8) China: Yunnan, Tengchong, *Wen, J.* *5680 (US)* | | 10 | 2 | 12 | 8 |
| (9) China: Yunnan, Baoshan, *Wen, J.* *6342 (US)* | | 10 | 2 | 12 | 8 |
| (10) China: Yunnan, Gaoligongshan, *Wen, J.* *13862 (US)* | | 10 | 2 | 12 | 8 |
| (11) China: Yunnan, Marlipo, *Wen, J.* *5627(10) (US)* | | 10 | 2 | 12 | 8 |
| (12) China: SE Yunnan, *Wen, J.* *10634 (US)* | | 10 | 2 | 12 | 8 |
| (13) China: SE Yunnan, *Shui, Y.M.* *81150.2.2 (US)* | | 10 | 2 | 12 | 8 |
| (14) China: SE Yunnan, *Wen, J.* *81097(212) (US)* | | 10 | 2 | – | – |
| (15) China: Yunnan, Maguan, *Wen, J.* *5580 (US)* | | – | 2 | 12 | – |
| (16) China: SW Yunnan, *Vargas, P.* *206PV04 (MAUAM)* | | 10 | 2 | 12 | 8 |
| (17) China: Xizang Province, Linzhi Xian, Dongjinxiang, Wen, J. *9172 (US)* | | – | 2 | – | – |
| (18) China: Zeilang, Chongqing, Mt. Jinyun, *Vargas, P.* *120PV03(1) (MAUAM)* | | 10 | 2 | 16 | 11 |
| (19) China: Jiangxi, Lushan, *Wen, J.* *5523 (US)* | | 10 | 2 | – | – |
| (20) China: Jiangxi, Lushan, Taiyicun, Min-xiang, N. *92028 (TNM S18974)* | | – | 2 | 12 | – |
| (21) Vietnam: Lao Cai, Fan-si Pan, *McAllister, H.A.* *895HAM (LIV)* | | – | 2 | 12 | – |
| (22) Vietnam: Lao Cai Province, Sa Pa District, Sa Pa, Ham Rong Mountain, *Wen, J. 5980 (US)* | | 10 | 2 | 12 | 8 |
| (23) Vietnam: Lao Cai Province, Sa Pa District, Sa Pa, Ham Rong Mountain, *Wen, J. 10899 (US)* | | – | 2 | 12 | – |
| (24) Vietnam: Lao Cai Province. Sa Pa District, Sa Xeng Village, *Wen, J. 6083 (US)* | | 10 | 2 | – | – |
| (25) Vietnam, *KR2884 (LIV)* | | – | 2 | 12 | – |
| ***Hedera pastuchowii*** Woronow subsp***. cypria*** (McAllister) Hand | **Western Mediterranean: Cyprus** | | | | |
| (1) Cyprus: Limasol, Apsein, *McAllister, H.A. 504HAM (LIV)* | | – | – | 10 | – |
| (2) Cyprus: Troodos Mountains, Kakopetria, *McAllister, H.A.* *188HAM (LIV)* | | – | – | 6 | – |
| (3) Cyprus: Prodromus - Kakopetria, *Valcárcel, V.* *07VV05(4) (MAUAM)* | | 2 | 5 | 6 | 15 |
| (4) Cyprus: Platres, *Valcárcel, V.* *12VV05(11) (MAUAM)* | | 2 | 5 | 6 | 15 |
| ***Hedera pastuchowii*** Woronow subsp***. pastuchowii*** | **South Caucasus, Iran and Afghanistan** | | | | |
| (1) Afghanistan: Kunar, Dewagal Darrah, Umgebung von Chalas, *Anders, O.* *11052 (MSB01495)* | | 10 | – | – | – |
| (2) Caucasus: Cultivated in Edinburgh from Caucasus material, *(MAUAM)* | | 10 | 2 | 14 | 12 |
| (3) Iran: Elburz mountains, *McAllister, H.A.* *259HAM (LIV)* | | – | – | 12 | – |
| (4) Iran: Mazanderan, Talair valley, *Aramshad, Zumer, M.* *74bMZ (BG)* | | – | 2 | – | – |
| (5) Iran: Haraz, Valley Kareshang, *Wendelbo, P.* *s.n. (1950BG)* | | – | 2 | – | – |
| ***Hedera rhombea*** (Miq.) Bean | **E Asia: Japan, Korea and Taiwan** | | | | |
| (1) Japan: Ishikawa Pref. *Fujii 7390F (5290MAKINO)* | | – | – | 12 | – |
| (2) Taiwan: Nagano, *Asano, K & Asano, H.* *s.n. (336811MAKINO)* | | – | – | 13 | – |
| (3) Japan: Natou Hsien, Jenai Hsiang, Junghsing Village, *Yang, T.Y.A.* *et al.* *11706 (TNM S70029)* | | 11 | 2 | 12 | 9 |
| (4) Japan: Oita, usa-jingir, *Esténabez, B.* *s.n. (MAUAM)* | | – | 2 | 12 | – |
| (5) Japan: Shizuoka, Fukuroi-shi, Mt. Ogasayama, *Tadashi, Y.* *3765YT (TAIPEI 096390TAIF)* | | – | 2 | 12 | – |
| (6) Japan: Shizouka Pref., Kamo-gun, Higashi izu-cho, along the Kawakubo-kawa, *Murata, J.* *et al. s.n. (M03-01-57TI)* | | – | 2 | – | – |
| (7) Japan: Tokyo, Mt Takanose, *K. Suzuki 9676 (270910MAKINO)* | | – | – | 12 | – |
| (8) Korea: South coast, *S. Lee s.n. (LIV)* | | – | – | 6 | – |
| (9) Korea: Ulluengdo Island, *Baek, W.K. s.n. (0013651GANGWON)* | | 10 | 2 | – | – |
| (10) Taiwan: Chiayi, Sheau Tashan, *Hwang, Y.H. & Chen, S.J.* *s.n. (TNM S9202)* | | – | – | 1 | – |
| (11) Taiwan: Taipei Tatunshan, *Chian 3308 (180551TAIF)* | | – | – | 12 | – |
| (12) Taiwan: Taipei, *Wang, C.M. & Tsai, Y.H.* *03011 (TNM S48777)* | | – | – | 12 | – |
| (13) Taiwan: Taipei, Tansmingshan National Park, *McAllister, H.A.* *869HAM (LIV)* | | 10 | 1 | 12 | 7 |
| (14) Taiwan: Taichung, *Lii 348 (152957TAIF)* | | – | – | 12 | – |
| (15) Taiwan: Taichung Hsien, Hoping Hsiang, *Shen, H.Y.* *et al. 415 (TNM S16067)* | | – | 2 | 12 | – |
| (16) Taiwan: Taoyuan, *Kuo, S.M. 206 (178186TAIPEI)* | | – | – | 12 | – |
| (17) Taiwan, *Liu s.n. (US)* | | – | 2 | 12 | – |
| (18) Taiwan: Miaoli Co., Wu Feng Hsiang, Kuanwu, *Chen, C.H.* *et al. 824 (TAIPEI 075074)* | | – | 2 | 2 | – |
| (19) Taiwan: Ilan County, Ssuyuan, *Cheng 3856 (TAIPEI 139079TAIF)* | | 10 | 2 | – | – |
